# Towards standardisation: comparison of five whole genome sequencing (WGS) analysis pipelines for detection of epidemiologically linked tuberculosis cases

**DOI:** 10.2807/1560-7917.ES.2019.24.50.1900130

**Published:** 2019-12-12

**Authors:** Rana Jajou, Thomas A Kohl, Timothy Walker, Anders Norman, Daniela Maria Cirillo, Elisa Tagliani, Stefan Niemann, Albert de Neeling, Troels Lillebaek, Richard M Anthony, Dick van Soolingen

**Affiliations:** 1 Tuberculosis Reference Laboratory, National Institute for Public Health and the Environment (RIVM), Bilthoven, the Netherlands; 2 Center of Epidemiology and Surveillance of infectious diseases, National Institute for Public Health and the Environment (RIVM), Bilthoven, the Netherlands; 3 These authors contributed equally; 4 Molecular and Experimental Mycobacteriology, Forschungszentrum Borstel, Borstel, Germany; 5 German Center for Infection Research, Borstel site, Borstel, Germany; 6 Nuffield Department of Medicine, University of Oxford, John Radcliffe Hospital, Oxford, United Kingdom; 7 International Reference Laboratory of Mycobacteriology, Statens Serum Institut, Copenhagen, Denmark; 8 Emerging Bacterial Pathogens Unit, Division of Immunology, Transplantation and Infectious Diseases, IRCCS San Raffaele Scientific Institute, Milan, Italy; 9 German Center for Infection Research (DZIF), Partner Site Hamburg-Lübeck-Borstel-Riems, Germany; 10 Global Health Section, Department of Public Health, University of Copenhagen, Copenhagen, Denmark

**Keywords:** Whole genome sequencing, analysis pipelines, international, epidemiology, tuberculosis, TB

## Abstract

**Background:**

Whole genome sequencing (WGS) is a reliable tool for studying tuberculosis (TB) transmission. WGS data are usually processed by custom-built analysis pipelines with little standardisation between them.

**Aim:**

To compare the impact of variability of several WGS analysis pipelines used internationally to detect epidemiologically linked TB cases.

**Methods:**

From the Netherlands, 535 *Mycobacterium tuberculosis* complex (MTBC) strains from 2016 were included. Epidemiological information obtained from municipal health services was available for all mycobacterial interspersed repeat unit-variable number of tandem repeat (MIRU-VNTR) clustered cases. WGS data was analysed using five different pipelines: one core genome multilocus sequence typing (cgMLST) approach and four single nucleotide polymorphism (SNP)-based pipelines developed in Oxford, United Kingdom; Borstel, Germany; Bilthoven, the Netherlands and Copenhagen, Denmark. WGS clusters were defined using a maximum pairwise distance of 12 SNPs/alleles.

**Results:**

The cgMLST approach and Oxford pipeline clustered all epidemiologically linked cases, however, in the other three SNP-based pipelines one epidemiological link was missed due to insufficient coverage. In general, the genetic distances varied between pipelines, reflecting different clustering rates: the cgMLST approach clustered 92 cases, followed by 84, 83, 83 and 82 cases in the SNP-based pipelines from Copenhagen, Oxford, Borstel and Bilthoven respectively.

**Conclusion:**

Concordance in ruling out epidemiological links was high between pipelines, which is an important step in the international validation of WGS data analysis. To increase accuracy in identifying TB transmission clusters, standardisation of crucial WGS criteria and creation of a reference database of representative MTBC sequences would be advisable.

## Introduction

Since the early 1990s, several DNA typing methods for *Mycobacterium tuberculosis* complex (MTBC) isolates have been developed, such as IS*6110* restriction fragment length polymorphism typing [1], mycobacterial interspersed repeat unit-variable number of tandem repeat (MIRU-VNTR) typing [2] and spoligotyping [3]. Although these technologies have revolutionised the possibilities to study the epidemiology of tuberculosis (TB), they lack sufficient resolution and are often technically demanding, laborious and/or time-consuming. Whole genome sequencing (WGS) has gained increasing recognition as the new standard approach for epidemiological typing of MTBC. It has the highest resolution and an additional advantage in allowing for simultaneous identification of the MTBC (sub)species and genotype families [4,5], as well as detection of resistance to anti-tuberculous drugs in a reliable way [6,7]. Multiple studies regarding the epidemiology of TB have pointed out that the resolution of WGS is superior to that of MIRU-VNTR typing and that epidemiological links can be traced more accurately [8-16].

Due to the highly conserved genome of MTBC strains, it is possible to analyse WGS data from any MTBC strain by comparison to a common reference genome. The *M. tuberculosis* H37Rv genome has been widely used as a reference genome and mutations compared to the H37Rv genome are reported as single nucleotide polymorphisms (SNPs) or insertions and deletions. Nevertheless, the analysis of WGS data remains subject to variability due to the presence of repetitive regions in the genome that cannot be accurately analysed using the most widely applied WGS techniques. Therefore, genomic regions with repetitive sequences are generally excluded from data analysis. Currently, there is no international standardisation in the analysis of WGS data, e.g. for the exact genomic regions excluded, the applied software and parameters, or the quality and quantity required for sequence data.

Initially, a maximum distance of 12 SNPs between *M. tuberculosis* isolates was introduced to rule in a possible epidemiological link between TB cases [17]. However, this threshold is influenced by the stringency applied in the WGS analysis and the genetic diversity of MTBC strains in the area of interest [9]. A pilot project was initiated by the European Centre for Disease Prevention and Control (ECDC) in 2018 to evaluate the large-scale implementation of WGS across Europe in substitution of MIRU-VNTR typing [18].

Core genome multilocus sequence typing (cgMLST) has been suggested as an alternative for genotyping of MTBC strains from WGS data, using a set of 2,891 genes that can be reliably recovered from the WGS data for any MTBC strain [19,20]. Similar to the SNP-based approach, a difference in more than 12 alleles of the scheme has been suggested as the threshold to rule out recent transmission [20].

In this study, we compare the analysis of WGS data of 535 culture-positive MTBC isolates from the Netherlands; data analysis was performed at four different European institutes: National Institute for Public Health and the Environment (RIVM; Bilthoven, the Netherlands), Oxford University (Oxford, United Kingdom), Research Center Borstel (Borstel, Germany) and Statens Serum Institut (SSI; Copenhagen, Denmark), using four distinct SNP-based analysis pipelines and a cgMLST gene-by-gene approach. Results of the five individual WGS pipelines were compared regarding their ability to rule out an epidemiological link between TB cases.

## Methods

### Whole genome sequencing dataset from the Netherlands

In total, 535 routinely collected culture-positive MTBC isolates from the Netherlands in 2016 were subjected to MIRU-VNTR typing [2,21] and WGS (see Supplementary Table S1 for 24-loci MIRU-VNTR classification and Supplementary Table S2 for sequence quality). DNA used for sequencing was isolated from positive Mycobacteria Growth Indicator Tubes and purified with the QIAamp DNA mini kit method (QIAGEN GmbH, Hilden, Germany). Libraries were prepared using the Nextera XT DNA Library Prep Kit and run on the Illumina HiSeq 2500 sequencer that generated 2 x 125bp paired-end reads. In order to achieve a mean coverage depth of ≥ 80 reads, a minimum sample yield of 350 Mbp was required for sequenced samples considering the 4.4 Mbp genome size of *M. tuberculosis*.

Of 535 cases, 134 were clustered with another case in 2016 by MIRU-VNTR typing (i.e. patient isolates sharing identical 24-loci patterns) and epidemiological investigation was performed by MHSs for these cases [22]. The MHSs assessed whether transmission was likely between the 134 clustered patients using information they obtained during interviews with the patients over several months. As described in the original study [22], the 134 MIRU-VNTR clustered cases belong to 41 different MIRU-VNTR clusters, where cluster sizes range from 2–21 isolates and 25 of 41 clusters consisted of two isolates. Anonymised patient characteristics, e.g. age, sex, ethnicity and risk group, for all cases were obtained from the Netherlands Tuberculosis Register [23]. This register contains patient characteristics, laboratory results, results of source-and contact tracing and information regarding the diagnosis and treatment of all TB patients and latent TB cases from the Netherlands. In the original study [22], PhyResSe [24] was used to assign lineages to the *M. tuberculosis* isolates. In the Netherlands, 75% of TB cases are foreign-born [25] and all major phylogenetic lineages of *M. tuberculosis* are represented in this set of isolates.

The 535 strains with RIVM sample numbering were first coded into unique sample numbers for each institute. Sequence data for the strains, generated at the RIVM, were then shared in fastq format with the Research Center Borstel and Oxford University; SSI downloaded reference mapped reads in Bam format from the European Nucleotide Archive (accession number PRJEB25592). WGS data for the 535 TB cases were analysed using a cgMLST-based gene-by-gene approach with the commercially available software SeqSphere+ and four different in-house SNP-based analysis pipelines from the Oxford University, Research Center Borstel, SSI and RIVM. Of these, the SNP pipeline from Borstel has been published under the name MTBseq [26].

Each institute ran the sequence data using their respective in-house developed WGS analysis pipeline(s) and were blinded to the results of cluster investigations, i.e. whether patients were epidemiologically linked according to the investigations by the MHSs. A summary of settings applied in each SNP-based pipeline can be seen in Table 1. All institutes provided a genetic distance matrix of the complete dataset that passed the quality metrics and, in addition, quality metrics for the datasets excluded from the analysis due to lack of sufficient sequence quality. The combined results were analysed at the RIVM using R version 3.3.2 (R foundation for statistical computing, Vienna, Austria).

**Table 1:** Summary of whole genome sequencing pipeline settings applied for each SNP pipeline

Settings	RIVM SNP	Oxford University SNP	Research Center Borstel (MTBseq) SNP	SSI SNP
H37Rv reference genome version	3	2	3	3
Alignment software	Bowtie	Stampy	BWA	BWA
SNP calling software	Breseq	Samtools	Samtools	Samtools
Minimum mean sample coverage depth	≥ 20x	NA	≥ 30x	≥ 20x
Minimum sample coverage breadth	NA	> 88%	≥ 80% fulfilling thresholds for variant detection	≥ 95%
Genomic regions excluded	Repeats	Repeats	Repeats, resistance genes	Repeats
Minimum coverage depth to support a SNP	NA	5x (one forward, one reverse, < 10% alternative allele)	8x (four forward, four reverse, four with phred score ≥ 20)	8x (four forward, four reverse)
Excluding SNPs within 12bp	Yes	No	Yes	Yes
Allele frequency	≥ 80%	≥ 90%	≥ 75%	≥ 85%
Dealing with low coverage positions or positions not meeting variant call criteria when calculating the genetic distance	Report reference base	Report consensus base	Report consensus base or exclude position if data quality is below thresholds in >5% of samples	Complement with data from aligned reads if coverage is > 5x or exclude position if data quality is below threshold

BWA: Burrows-Wheeler Alignment; MTB: *Mycobacterium tuberculosis*; NA: not applicable; RIVM: National Institute for Public Health and the Environment; SNP: single nucleotide polymorphism; SSI: Statens Serum Institut.

The distance matrices from each participating institute were sent to the RIVM. Using R, the matrices from each institute were decoded into the RIVM sample numbering, transformed to distance matrices in the long format and then merged together so that the results of the distinct pipelines could be compared more efficiently. Following this, a subset was created for isolates that were clustered by WGS for each pipeline; a pairwise genetic distance of 12 SNPs/alleles was used as threshold for clustering of cases by WGS [17,20]. We investigated whether strains clustered by MIRU-VNTR that had been isolated from patients with confirmed epidemiological links were also clustered by WGS.

### The SNP pipeline from the National Institute for Public Health and the Environment

Fastq.gz files were mapped unpaired against the H37Rv reference genome (GenBank accession: AL123456.3) using Bowtie2 in Breseq version 0.28.1 [27]. Sequences with a mean sample coverage depth below 20x were excluded from the data analysis. For isolates with a sufficient coverage depth, an allele frequency of ≥ 80% was applied to detect SNPs, for which Breseq produced a Genome Difference file that included all SNPs. This Genome Difference file was imported into R for further analysis: excluding genetic regions annotated as PE/PPE, PGRS, pks, esx, repeat, polyketide or transposase in the gene product description of the annotated Genome Difference file. In addition, positions of repeat regions as annotated in the GenBank H37Rv reference genome and all SNPs within 12bp apart from each other were excluded as well. This set of SNPs was used to compute pairwise genetic distances using ‘ape’ and ‘phangorn’ packages from R. The SNP pipeline from the RIVM can be found in Supplementary Material S1.

### The SNP pipeline from Oxford University

Sequence read data were mapped to the H37Rv reference genome (GenBank accession: NC_000962.2) using Stampy version 1.0.17 (without BWA pre-mapping, using an expected substitution rate of 0.01) [28]. SNPs were identified across all mapped non-repetitive sites using Samtools mpileup version 1.0.18 [29]. Repetitive sites were identified using self-self BLAST of 75bp reference genome reads and excluded from further analysis. Only SNPs supported by at least five high quality reads, including one in each direction, were accepted and at least 75% of reads were required to be of high quality. Calls were required to be homozygous under a diploid model, with mixed calls assigned where a minority allele composes > 10% of read depth. The SNP pipeline from the Oxford University is accessible via https://github.com/oxfordmmm/CompassCompact. The docker image is available via: https://cloud.docker.com/u/oxfordmmm/repository/docker/oxfordmmm/compasscompact.

### The SNP pipeline from the Research Center Borstel (MTBseq)

Reads were aligned to the *M. tuberculosis* H37Rv genome (GenBank accession: NC_000962.3) with the alignment program BWA [30] and mappings refined with the GATK [31] and Samtools toolkits [29] for re-calibration, re-alignment and read deduplication. For variant detection in mapped reads for phylogenetic analysis, we employed MTBseq default values, i.e. Samtools mpileup output was filtered for minimum thresholds of four reads calling the allele in both forward and reverse orientation, four reads calling the allele with at least a phred score of 20 and a minimum of 75% allele frequency. Datasets with a mean coverage depth below 30x, less than 80% of the reference genome complying with minimum quality thresholds, and/or substantial contamination (inferred from less than 80% of reads mapped to the reference genome) were excluded from further analysis. For a joint phylogenetic comparison, detected variant positions were combined, complementing the joint list with detailed sequence information from the original mappings. After excluding variant positions appearing within a window of 12bp in the same isolate and positions in drug resistance-associated genes or repetitive regions [32], the remaining positions that match the minimum thresholds in at least 95% of all isolates and a valid base call in all isolates were used for a concatenated sequence alignment. Subpopulations within isolates were inferred from the genome-wide detection of low frequency variants, i.e. variants found in only a proportion of the sequence reads, as detected with the MTBseq low frequency modus. The MTBseq SNP pipeline from the Research Center Borstel is accessible via https://github.com/ngs-fzb/MTBseq_source [26].

### The core genome MLST approach (SeqSphere+)

WGS data in the form of BAM alignments created by the MTBseq pipeline were imported into the SeqSphere+ version 5.1.0 software (Ridom GmbH, Münster, Germany) and all genes defined as loci in the specified MLST scheme were extracted and the sequence submitted to the nomenclature server (cgMLST.org) for translation into allele numbers. The cgMLST scheme used contains 2,891 core genes and was defined using the MLST Target Definer tool of the SeqSphere+ program and a set of 45 strains covering the full known diversity of the MTBC [20]. Repetitive genes such as those from the PPE/PE-PGRS gene families are not included in the cgMLST scheme. For all genes contained in the cgMLST scheme, SeqSphere+ extracted the respective gene sequence from the BAM alignments, evaluated the sequence with its default quality metrics, and assigned allele numbers. Here, quality thresholds for valid allele calls include the rejection of a target if the length of target sequence does not equal the reference sequence length plus or minus three triplets, if there is any ambiguous base in the target consensus sequence (supported by less than 60% of reads) and if there is a ‘frameshift’ detected. Pairwise distances were calculated from the full set of 2,891 core genes, pairwise ignoring missing values for missing or rejected targets and excluding samples for which > 10% of cgMLST genes did not meet the quality criteria.

### The SNP pipeline from the Statens Serum Institut

Usually, reads are mapped to the H37Rv reference genome (GenBank accession: NC_000962.3) using the BWA mapping program [30] and refined using Samtools and picard toolkits, to remove PCR duplicates. However, as described above, for practical reasons the SSI analysed the mapped Bam files. Variants were called using the programs Samtools mpileup [29] and bcftools call, respectively. Basic raw variant filtering involves removing alleles with a phred (QUAL) score below 20 and a mapping depth (DP) below five. All repetitive regions of the H37Rv genome were excluded, such as transposases (IS-elements and transposons), tandem repeats and all members of the PPE-/PE-PGRS gene families. A core alignment of all samples was then generated using a perl script (vcf2fasta.pl) that first screens every sample for high-quality variant positions (i.e. four forward reads and four reverse reads, a minimum allele frequency > 85%, and a minimum of 12bp to the nearest neighbouring variant) and then adds all homozygous variant calls from all samples using these positions. Heterozygous variant calls were added as ambiguous bases (N). Furthermore, using the mapping depth measured by Samtools depth, positions with a mapping depth below five were masked out as Ns (gaps if depth is zero) in each sample. Finally, the alignment is screened for positions with universally conserved (monomorphic) alleles, or > 10% ambiguous base calls or gaps, which were then removed to produce the final core alignment. The SNP pipeline from the SSI is accessible via https://github.com/micronorman/IRLM-SnpPipeline.

## Results

In total, 527 of 535 TB cases had complete data from the Netherlands Tuberculosis Register, of which 40.8% (215/527) were in the age category 25–44 years (median: 35 years), 60% (316/527) were men and 78.4% (413/527) were first generation migrants. Ninety-seven percent (n = 520) of the isolates were *M. tuberculosis,* followed by 2.4% *M. bovis* (n = 13), 0.2% *M. caprae* (n = 1), and 0.2% *M. orygis* (n = 1). As part of the original study [22], the 520 *M. tuberculosis* isolates were assigned lineages using PhyResSe [24]; 127 isolates belonged to EAS, 117 to Delhi/CAS, 78 to Haarlem, 60 to EAI, 43 to Beijing, 57 to LAM, 12 to S-type, seven to Ural, four to Cameroon, three to Uganda, two to TUR, two to West African II, one to West African I and for seven isolates, no lineage could be assigned (Supplementary Table S3).

**Figure 1:**
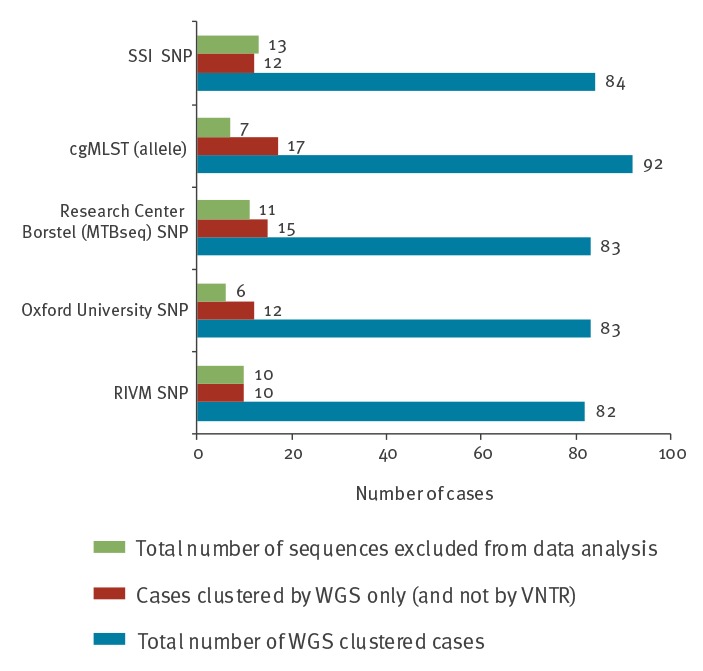
Clustering of cases by WGS in analysed samples using five distinct international WGS data analysis pipelines (n = 535)

A small proportion of the 535 datasets was excluded from the analysis in the individual pipelines due to poor sequence data quality: (i) 13 from the SSI pipeline, (ii) 11 from the Research Center Borstel pipeline (MTBseq), (iii) 10 from the RIVM pipeline, (iv) seven from the cgMLST approach, and (v) six from the Oxford University pipeline. The 10 samples excluded from the RIVM, Research Center Borstel, and SSI pipelines were not included in any further analysis (Supplementary Table S4, Figure 1). In addition, the Research Center Borstel (MTBseq) pipeline reported that 19 isolates were likely contaminated with non-mycobacterial DNA as inferred from the percentage of reads mapped to the H37Rv reference genome, however, these isolates were included in data analysis (see Supplementary Table S2).

cgMLST: core genome multilocus sequence typing; MTB: *Mycobacterium tuberculosis;* RIVM: National Institute for Public Health and the Environment; SNP: single nucleotide polymorphism; SSI: Statens Serum Institut; VNTR: variable number of tandem repeat*;* WGS: whole genome sequencing.

WGS clusters were defined using a maximum pairwise distance of 12 SNPs/alleles.

### Whole genome sequencing clustering by pipeline

Of the 535 isolates, 92 were clustered in the cgMLST-based approach, 84 in the SSI pipeline, 83 in the Oxford University pipeline, 83 in the Research Center Borstel (MTBseq) pipeline and 82 in the RIVM pipeline (Figure 1). In all pipelines, a small number of isolates were only clustered by WGS (and not MIRU-VNTR); this was highest in the cgMLST pipeline and lowest in the RIVM pipeline (17 and 10 isolates, respectively) (Figure 1). Almost all pairs of isolates that were clustered by WGS only varied by only one or two of the 24-MIRU-VNTR loci in their MIRU-VNTR profiles. One pair clustered by WGS only in the Oxford University pipeline, but showed up to 255 SNPs/allele difference by the four other pipelines (Table 2); the isolates in this pair may contain mixed populations, as indicated from the detection of low frequency variants by the Research Center Borstel (MTBseq) pipeline. Another two pairs of isolates were clustered by WGS only in the cgMLST approach, Oxford University pipeline and Research Center Borstel (MTBseq) pipeline, but not in the RIVM and SSI pipeline (Table 2). One of these paired isolates likely contained mixed bacterial populations, as indicated by the presence of low frequency variants as reported by the Research Center Borstel (MTBseq) pipeline.

**Table 2:** Genetic distances of pairs of isolates clustered by WGS only and not by MIRU-VNTR in five distinct international WGS data analysis pipelines and the associated 24-loci MIRU-VNTR patterns

Sample 1	Sample 2	Genetic distance in SNPs/alleles by pipeline					24-loci MIRU-VNTR order^a^	
		RIVM SNP	Oxford University SNP	Research Center Borstel (MTBseq) SNP	SSI SNP	cgMLST (allele)	MIRU-VNTR pattern sample 1	MIRU-VNTR pattern sample 2
ERX2465161	ERX2465207	12	14	12	8	8	2-5-3-5-3-3-2-3-3-4-1-3-6-3-**5**-2-5-2-2-1-3-4-2-3	2-5-3-5-3-3-2-3-3-4-1-3-6-3-**6**-2-5-2-2-1-3-4-2-3
ERX2465178^b^	ERX2465568^b^	14	12	8	5	5	2-1-4-7-4-**3**-4-2-4-2-2-4-2-3-5-2-5-3-2-1-3-4-2-3	2-1-4-7-4-**4**-4-2-4-2-2-4-2-3-5-2-5-3-2-1-3-4-2-3
ERX2465292	ERX2465259	17	19	16	15	12	2-**5**-1-3-3-3-2-**5**-3-2-6-2-5-2-5-1-6-2-2-1-3-4-2-3	2-**7**-1-3-3-3-2-**4**-3-2-6-2-5-2-5-1-6-2-2-1-3-4-2-3
ERX2465308	ERX2465278	16	15	11	9	12	2-5-2-**5**-4-5-3-2-4-2-**4**-4-2-4-7-2-5-3-2-1-3-4-2-3	2-5-2-**6**-4-5-3-2-4-2-**3**-4-2-4-7-2-5-3-2-1-3-4-2-3
ERX2465418^c,d^	ERX2465573	133	0	2	41	0	2-**5**-**2**-**6**-4-5-**3**-2-4-2-3-4-2-4-8-2-5-3-2-1-3-4-2-3	2-**4**-**3**-**5**-4-5-**2**-2-4-2-3-4-2-4-8-2-5-3-2-1-3-4-2-3
ERX2465418^c,d^	ERX2465330	132	0	3	41	1	2-**5**-**2**-**6**-4-5-**3**-2-4-2-3-4-2-4-8-2-5-3-2-1-3-4-2-3	2-**4**-**3**-**5**-4-5-**2**-2-4-2-3-4-2-4-8-2-5-3-2-1-3-4-2-3
ERX2465418^c,d^	ERX2465391^d^	123	0	255	189	185	2-5-**2**-**6**-4-5-3-2-**4**-2-3-4-2-**4**-8-2-5-3-2-1-3-4-2-3	2-5-**3**-**5**-4-5-3-2- **-2**-2-3-4-2-**3**-8-2-5-3-2-1-3-4-2-3
ERX2465512	ERX2465223	7	7	6	4	4	2-5-4-3-1-**3**-2-4-3-2-3-2-3-2-2-2-5-2-2-1-3-4-2-3	2-5-4-3-1-**4**-2-4-3-2-3-2-3-2-2-2-5-2-2-1-3-4-2-3
ERX2465622^b^	ERX2465178^b^	6	6	4	3	4	2-1-4-7-4-**4**-4-2-4-2-2-4-2-3-5-2-5-**4**-2-1-3-4-2-3	2-1-4-7-4-**3**-4-2-4-2-2-4-2-3-5-2-5-**3**-2-1-3-4-2-3
ERX2465622^b^	ERX2465568^b^	10	8	6	4	5	2-1-4-7-4-4-4-2-4-2-2-4-2-3-5-2-5-**4**-2-1-3-4-2-3	2-1-4-7-4-4-4-2-4-2-2-4-2-3-5-2-5-**3**-2-1-3-4-2-3
ERX2465631^e^	ERX2465366	1	0	0	0	0	2-6-2-7-3-4-2-3-3-4-7-3-2-**2**-7-2-5-2-2-1-3-4-2-2	2-6-2-7-3-4-2-3-3-4-7-3-2-**6**-7-2-5-2-2-1-3-4-2-2
ERX2465631^e^	ERX2465636	2	1	1	2	0	2-6-2-7-3-4-2-3-3-4-7-3-2-**2**-7-2-5-2-2-1-3-4-2-2	2-6-2-7-3-4-2-3-3-4-7-3-2-**6**-7-2-5-2-2-1-3-4-2-2

MIRU-VNTR: mycobacterial interspersed repeat unit-variable number of tandem repeat; MTB: *Mycobacterium tuberculosis*; RIVM: National Institute for Public Health and the Environment; SNPs: single nucleotide polymorphisms; SSI: Statens Serum Institut; WGS: whole genome sequencing.

^a^ The 24-loci MIRU-VNTR order was 580-2996-802-960-1644-3192-424-577-2165-2401-3690-4156-2163b-1955-4052-154-2531-4348-2059-2687-3007-2347-2461-3171.

^b^ This isolate clustered by WGS only with two isolates belonging to two different MIRU-VNTR clusters.

^c^ This isolate clustered by WGS only with three isolates belonging to two different MIRU-VNTR clusters.

^d^ This isolate likely contains subpopulations due to the presence of low frequency variants.

^e^ This isolate clustered by WGS only with two isolates belonging to the same MIRU-VNTR cluster.

Variation in the 24-loci MIRU-VNTR patterns between the pairs of isolates are bold and underlined.

### Association between genetic distance and epidemiological links

**Figure 2:**
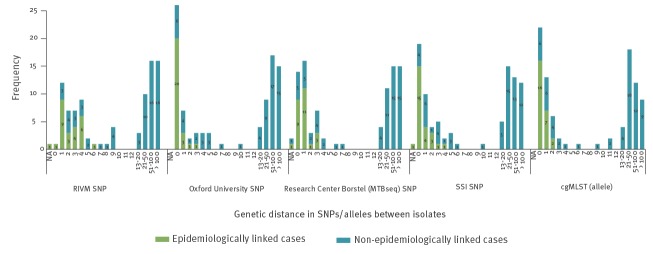
Association between the pairwise genetic distance and epidemiological links for the 134 MIRU-VNTR clustered tuberculosis cases, by WGS pipeline

Of the 535 isolates analysed, 134 were clustered with another isolate by MIRU-VNTR, and epidemiological cluster investigations were performed by MHSs for these MIRU-VNTR clustered cases. Results of cluster investigations showed that epidemiological links were identified for 41/134 MIRU-VNTR clustered cases and for the remaining 93 cases, no epidemiological links within the Netherlands could be identified. All pipelines exhibited short pairwise genetic distances between isolates of the 41 epidemiologically linked cases (Figure 2).

cgMLST: core genome multilocus sequence typing; MIRU-VNTR: mycobacterial interspersed repeat unit-variable number of tandem repeat; MTB: *Mycobacterium tuberculosis*; RIVM: National Institute for Public Health and the Environment; SNP: single nucleotide polymorphism; SSI: Statens Serum Institut; WGS: whole genome sequencing.

WGS clusters were defined using a maximum pairwise distance of 12 SNPs/alleles.

All 41 epidemiologically linked cases were clustered by the Oxford University SNP pipeline and the cgMLST approach using a threshold of ≤ 12 SNPs/alleles. However, one of these linked isolates had a relatively low mean coverage depth (13x), which was handled differently in each pipeline depending on the minimum mean sample coverage depth accepted for inclusion in the data analysis. As the RIVM, Research Center Borstel (MTBseq) and SSI pipeline applied a minimum mean sample coverage depth of 20x, 30x and 20x, respectively (Table 1), this isolate was excluded from data analysis. In the Oxford University pipeline, results showed a genetic distance of zero SNPs for this epidemiologically linked pair and the cgMLST approach reported a genetic distance of one allele. The mean (range) genetic distance in SNPs/alleles between the 41 epidemiologically linked cases was 2.4 (0–6), 0.3 (0–3), 0.9 (0–3), 0.4 (0–2) and 0.7 (0–4) in the RIVM, Oxford University, Research Center Borstel (MTBseq), cgMLST approach and SSI pipeline, respectively (Table 3).

**Table 3:** Results from five distinct international whole genome sequencing data analysis pipelines for the 134 isolates clustered by MIRU-VNTR with (n = 41) and without (n = 93) epidemiological link

Pipeline	WGS clustered (≤ 12 SNPs/alleles)		Non-WGS clustered (> 12 SNPs/alleles)		NA^a^	Genetic distance in SNPs/alleles by pipeline, mean (range)	
	Epidemiological link (Yes)	Epidemiological link (No)	Epidemiological link (Yes)	Epidemiological link (No)		Epidemiologically linked cases	Non-epidemiologically linked cases
RIVM SNP	39^b^	34	0	59	2	2.4 (0–6)	65.9 (0–198)
Oxford University SNP	41	34	0	59	NR	0.3 (0–3)	63.6 (0–209)
Research Center Borstel (MTBseq) SNP	39^b^	32^b^	0	59	4	0.9 (0–3)	55.7 (0–174)
cgMLST (allele)	41	39	0	54	NR	0.4 (0–2)	42.5 (0–132)
SSI SNP	39^b^	34	0	59	2	0.7 (0–4)	46.6 (0–151)

cgMLST: core genome multilocus sequence typing; NA: not applicable; NR: not recorded; MTB: *Mycobacterium tuberculosis*; RIVM: National Institute for Public Health and the Environment; SNP: single nucleotide polymorphism; SSI: Statens Serum Institut.

^a^ Not applicable for analysis since paired isolates were excluded due to low mean coverage depth.

^b^ One paired isolate was excluded due to low mean coverage depth.

Among the 93 patient isolates clustered by MIRU-VNTR, for which no epidemiological links were identified by MHSs, the cgMLST approach clustered 39 cases. The remaining pipelines all clustered the same 34 non-epidemiologically linked cases, except for one non-epidemiological link between two cases that was not analysed by the Research Center Borstel (MTBseq) pipeline due to low coverage (Table 3). The mean (range) genetic distance in SNPs/alleles for the 93 non-epidemiologically linked, MIRU-VNTR clustered cases was 65.9 (0–198), 63.6 (0–209), 55.7 (0–174), 42.5 (0–132) and 46.6 (0–151) in the RIVM, Oxford University, Research Center Borstel (MTBseq), cgMLST approach and the SSI pipeline, respectively (Table 3).

## Discussion

A representative set of 535 routine MTBC samples from the Netherlands was used to compare five different WGS data analysis pipelines. The five pipelines yielded highly comparable results, with only two epidemiologically linked cases being missed using the previously proposed threshold of 12 SNPs/alleles [17,20]. All *M. tuberculosis* lineages were represented in the dataset used in this study [22,25], meaning that the results could be extrapolated to other countries with a similar TB situation as the Netherlands, i.e. low TB incidence and low prevalence of drug resistance. In addition, it is possible that the dataset could be utilised for the evaluation of emerging WGS-based pipelines in the future. While the functionality of the pipelines will be similar in different settings, the usefulness of the SNP/allele threshold might be less applicable in countries with a high TB incidence where the TB population is more complex due to high prevalence of MDR-TB and/or mixed infections, e.g. India, China, Russia or South Africa [33-37]. In these high TB incidence countries, a static SNP/allele threshold may be less applicable if we assume that TB transmission between patients is more dynamic, long lasting and more challenging to interrupt, thus leading to large clonal clusters of nearly identical indirectly connected isolates. Advanced sequence techniques like MinION and PacBio that allow the complete *M. tuberculosis* genome (including the currently excluded repetitive regions) [38] to be analysed would be favourable, as this would allow for improved resolution to study TB transmission. Alternatively, approaches have been suggested that combine metadata such as timing or epidemiological information with genome data to infer possible transmission events [9,39-42].

All pipelines clustered additional cases for which no epidemiological links were identified by epidemiological investigations. Interestingly, all pipelines detected a few isolates that were clustered by WGS only (but not by MIRU-VNTR). As epidemiological data was only collected for patients clustered by MIRU-VNTR, epidemiological linkage information was not available for the cases clustered by WGS only. These cases could, however, represent actual transmission chains that were missed by MIRU-VNTR due to one or two repeat number changes during transmission from patient to patient. On occasion, MHSs find an epidemiological link between cases, e.g. a mother and daughter, that could not be genotypically confirmed as they do not have identical MIRU-VNTR patterns. These might represent the few patient isolates from our study that were found to be clustered by WGS only. The few discrepant results between pipelines in the clustering of isolates by WGS only were most likely due to the presence of mixed bacterial populations, which were handled differently depending on the settings applied by each pipeline. For example, in the two pairs clustered by WGS only in the cgMLST approach, Oxford University pipeline and Research Center Borstel (MTBseq) pipeline, one of the isolates contained two clonal populations with varying MIRU-VNTR patters in 4/24 loci. The SNPs that caused the genetic differences between these pairs were present at lower allele frequencies. Isolates containing mixed populations or contaminating DNA are likely under-reported due to the detection of mixed infections is not widely implemented in analysis pipelines. Therefore, more detailed investigation into this issue would be warranted to help guide the international standardisation of WGS analysis.

The genetic distances between strains of MIRU-VNTR clustered cases were in general lowest in the cgMLST approach and highest in the RIVM SNP pipeline – and consequently most cases were clustered by the cgMLST approach and fewest cases by the RIVM pipeline. While the focus of the current study was to investigate whether distinct pipelines were able to identify the epidemiologically linked cases, we did observe an unexpected result for the highly unrelated strains that were not clustered by WGS or MIRU-VNTR. The cgMLST approach, for obvious reasons, showed relatively lower genetic distances especially for highly unrelated strains. However, among the SNP-based pipelines, the less stringent RIVM pipeline (with respect to the allele frequency, the minimum coverage depth to support a SNP and dealing with low coverage positions when calculating the genetic distance) sometimes showed lower genetic distances between highly unrelated strains compared to the more stringent Oxford University SNP pipeline. A possible explanation for this could be that the RIVM pipeline excludes all SNPs within 12bp apart, while this rule is not applied in the Oxford University pipeline. These differences do not have an impact on the identification of epidemiologically linked cases, but could be studied in more detail to better understand the characteristics of the different pipelines.

The same isolates clustered by WGS in the RIVM SNP pipeline were also clustered in the four other pipelines, however, more non-epidemiologically linked cases were clustered in the other four pipelines. It is possible that these cases represent actual transmission missed by MHSs, as cluster investigation by interviews also misses a proportion of epidemiological links [43]. On the contrary, it may be that the genetic diversity of circulating strains is too low to rule out transmission even if zero SNPs are identified by the current WGS analysis pipelines [17,44]. Several previous studies reported relatively small genetic distances (≤ 12 SNPs) between non-epidemiologically linked cases [13,15,17,45], meaning a higher proportion of non-epidemiologically linked cases would be falsely clustered by WGS in the more stringent pipelines.

Several differences were observed between WGS pipelines with respect to the applied parameters, which can explain the few discrepant results between pipelines in the exclusion of sequence data from data analysis and the clustering of cases by WGS. These pipeline differences can be divided into four levels: (i) alignment, (ii) quality metrics, (iii) SNP calling, and (iv) distance calculation. First, the different pipelines applied distinct programs for alignment against the H37Rv reference genome. Second, pipelines applied varied quality criteria with respect to the minimum coverage depth (e.g. 20x, 30x) and breadth, which led to differences in the number of datasets excluded from data analysis as shown in this study. However, this does not influence the pairwise genetic distances calculated for each included isolate. Third, on the SNP calling level different criteria were applied that partly explain the differences in pairwise genetic distances reported by each pipeline. One major difference lies in the genetic regions excluded during data analysis, which consisted of excluding repetitive regions only, excluding drug resistance associated genes in addition, and/or excluding SNPs within 12bp in addition. Furthermore, differences were observed in the minimum coverage depths applied to support a SNP (e.g. 5x, 8x), minimum allele frequencies (e.g. 75%, 80%, 85%), and the software used for SNP calling. Finally, pipelines behave differently in how positions not meeting respective thresholds are treated when calculating the genetic distance. One option is to treat these positions as the reference sequence, another option is to exclude these regions from the whole database in case they are missing in at least one isolate and a third option is to capture the respective sequence information, either by using a consensus approach or by complementing the respective positions with data from aligned reads.

It is striking and encouraging that despite differences in the pipelines, for identification of highly epidemiologically related strains the performance was generally similar. The absolute number of SNPs called between more distant isolates showed more variability but was not the focus of this comparison. We believe that differences in the SNP calling parameters (e.g. excluded genetic regions, minimum coverage to support a SNP, allele frequency) and how to deal with positions with missing or low quality sequence data when calculating the genetic distance mainly explain the differences observed between pipelines. Better understanding of the individual pipeline characteristics and limitations is needed in order to accurately interpret and compare results between the pipelines and to set standards for the implementation and reporting of WGS data analysis.

### Conclusion

Although different approaches were taken to analyse WGS data, all pipelines were able to clearly distinguish epidemiologically linked cases from highly unrelated cases. Standardisation on crucial criteria of WGS data analysis and reporting on an international level will allow more efficient investigations of cross-border transmission and will help establish protocols allowing inter-laboratory comparison of results. To allow routine identification and monitoring of the spread of specific clusters, a database of strains [18] or a depository of cluster type strain sequence data or a similar approach is required. Having a representative set of sequence data that includes all MTBC (sub)species and (sub)lineages publicly available in an international database, would allow better understanding of the circulating and spread of strains. Finally, it is crucial to link genome and epidemiological data in order to study TB transmission with more resolution.

## Supplementary Data

999000 Supplementary Tables S1-S4

## Supplementary Data

463000 Supplementary Material S1
