# Supplementary material for: Towards standardisation: comparison of five whole genome sequencing (WGS) analysis pipelines for detection of epidemiologically linked tuberculosis cases
Source: Euro Surveill. 2019 Dec 12;24(50):1900130. doi: 10.2807/1560-7917.ES.2019.24.50.1900130 (PMC6918587; doi:10.2807/1560-7917.ES.2019.24.50.1900130)
Supplement: Supplementary Tables S1-S4 [file 19-00130_SupplementaryTablesS1-S4.pdf]

Supplementary Tables S1-S4

This supplementary material is hosted by *Eurosurveillance* as supporting information alongside the article [Towards standardisation: comparison of five whole genome sequencing (WGS) analysis pipelines for detection of epidemiologically linked tuberculosis cases], on behalf of the authors, who remain responsible for the accuracy and appropriateness of the content. The same standards for ethics, copyright, attributions and permissions as for the article apply. Supplements are not edited by *Eurosurveillance* and the journal is not responsible for the maintenance of any links or email addresses provided therein.

Supplementary Table S1. 24-loci MIRU-VNTR classification of the 535 *M. tuberculosis* complex isolates. Five isolates are represented in duplicate as they had double alleles at one VNTR loci and therefore two distinct 24-loci MIRU-VNTR patterns. This supplementary material is hosted by *Eurosurveillance* as supporting information alongside the article [Towards standardisation: comparison of five whole genome sequencing (WGS) analysis pipelines for detection of epidemiologically linked tuberculosis cases], on behalf of the authors, who remain responsible for the accuracy and appropriateness of the content. The same standards for ethics, copyright, attributions and permissions as for the article apply. Supplements are not edited by *Eurosurveillance* and the journal is not responsible for the maintenance of any links or email addresses provided therein.

strain	VNTR580	VNTR2996	VNTR802	VNTR960	VNTR1644	VNTR3192	VNTR424	VNTR577	VNTR2165	VNTR2401	VNTR3690	VNTR4156	VNTR2163b	VNTR1955	VNTR4052	VNTR154	VNTR2531	VNTR4348	VNTR2059	VNTR2687	VNTR3007	VNTR2347	VNTR2461	VNTR3171
ERX2465512	2	5	4	3	1	3	2	4	3	2	3	2	3	2	2	2	5	2	2	1	3	4	2	3
ERX2465208	2	6	3	5	3	3	2	3	3	3	3	3	4	2	7	2	3	2	2	1	3	2	2	3
ERX2465608	3	2	2	4	3	4	1	4	4	2	2	1	9	10	5	2	6	3	2	2	3	3	6	3
ERX2465437	2	4	3	5	4	5	4	2	4	2	3	4	2	4	8	2	5	3	2	1	3	4	2	3
ERX2465317	4	2	3	4	3	5	1	4	9	2	4	1	4	6	4	2	6	3	2	2	1	3	3	3
ERX2465584	2	5	4	4	3	3	4	3	3	4	3	3	3	3	5	2	5	2	2	1	3	4	2	3
ERX2465264	2	6	3	5	3	3	2	3	3	3	3	3	4	2	7	2	3	2	2	1	3	2	2	3
ERX2465292	2	5	1	3	3	3	2	5	3	2	6	2	5	2	5	1	6	2	2	1	3	4	2	3
ERX2465384	2	5	5	3	1	3	2	4	2	2	3	2	4	2	2	2	5	2	2	1	3	4	2	3
ERX2465263	2	4	2	3	4	3	2	4	2	2	3	2	5	2	4,5	2	5	2	2	1	1	4	2	2
ERX2465424	4	2	3	4	3	5	2	3	5	2	4	1	7	6	5	2	6	3	2	2	3	3	3	3
ERX2465507	5	2	2	4	3	4	1	2	4	2	2	1	9	11	6	2	6	3	2	2	3	3	6	3
ERX2465312	4	5	2	2	3	3	4	4	-2	2	1	2	-2	1	8	2	4	2	2	1	2	4	2	3
ERX2465675	2	5	3	4	3	3	2	3	3	4	3	3	5	3	5	2	5	2	2	1	3	4	2	3
ERX2465673	5	1	2	4	2	5	2	4	7	2	2	1	7	8	7	2	6	2	2	2	3	3	5	3
ERX2465314	2	5	1	3	3	3	2	4	3	2	5	2	3	2	4	2	6	2	2	1	3	4	1	3
ERX2465525	5	1	3	4	3	6	2	4	6	1	4	1	3	8	6	2	10	3	2	2	3	3	4	3
ERX2465411	5	1	2	4	2	5	2	4	7	2	2	1	7	8	7	2	6	2	2	2	3	3	5	3
ERX2465180	3	5	1	3	3	2	3	4	1	1	2	2	2	3	6	1	6	2	2	1	3	4	2	3

ERX2465481	2	6	4	4	3	3	2	3	3	4	3	3	3	2	7	2	5	2	2	1	3	4	2	3
ERX2465591	2	5	3	6	4	5	3	2	4	2	3	4	2	4	7	2	5	3	2	1	3	4	2	3
ERX2465293	5	1	2	4	2	5	2	4	7	2	2	1	7	8	7	2	6	2	2	2	3	3	5	3
ERX2465635	2	5	1	4	3	3	4	4	2	2	2	2	3	2	6	2	6	2	2	1	3	4	2	3
ERX2465441	2	1	2	10	2	2	2	4	4	4	3	3	2	3	8	2	5	2	2	1	3	4	2	3
ERX2465441	2	1	2	9	2	2	2	4	4	4	3	3	2	3	8	2	5	2	2	1	3	4	2	3
ERX2465587	2	5	3	5	3	3	2	3	3	3	3	2	6	2	7	2	3	2	1	1	3	2	2	3
ERX2465503	2	8	5	3	3	3	2	4	3	2	4	0	3	2	5	2	6	2	2	1	3	4	2	3
ERX2465399	5	2	3	4	3	6	2	4	6	1	4	1	3	11	6	2	10	3	2	2	3	3	4	3
ERX2465593	2	4	2	2	2	4	2	4	3	2	1	2	4	2	6	2	3	2	1	1	3	4	2	3
ERX2465373	2	5	3	3	2	3	2	2	3	2	2	2	2	2	6	2	5	2	2	1	4	4	2	3
ERX2465323	5	2	3	4	2	5	2	4	5	2	4	1	2	5	6	2	5	3	2	2	3	3	3	3
ERX2465571	5	2	2	4	3	4	1	4	4	2	2	1	9	10	4	2	6	2	2	2	3	3	6	3
ERX2465239	2	5	3	6	4	5	3	2	4	2	3	4	2	4	7	2	5	3	2	1	3	4	2	3
ERX2465304	2	7	3	4	3	4	5	4	4	4	3	2	5	5	9	2	5	3	2	1	3	4	2	3
ERX2465610	2	5	3	6	4	5	3	2	4	2	3	4	2	4	9	2	5	3	2	1	3	4	2	3
ERX2465228	2	1	2	10	2	3	3	4	4	3	3	3	2	3	8	2	5	2	2	1	3	4	2	3
ERX2465589	5	2	2	4	2	3	2	4	7	2	2	1	2	2	6	2	6	2	2	2	3	3	5	3
ERX2465187	2	5	4	4	2	3	4	2	2	2	1	2	3	2	6	2	6	1	2	1	3	4	2	1
ERX2465283	2	4	1	6	2	5	2	6	4	4	4	3	3	3	6	2	4	2	2	2	3	3	4	3
ERX2465664	2	7	3	3	3	1	4	4	4	4	3	2	5	5	8	2	5	3	2	1	3	4	2	3
ERX2465579	2	5	3	5	3	3	2	3	3	4	3	3	4	3	7	2	5	2	2	1	3	4	2	3
ERX2465405	2	5	1	3	2	3	1	4	2	1	2	2	2	3	4	2	6	2	2	1	3	5	2	3
ERX2465470	3	3	2	6	2	5	2	5	4	4	4	3	4	3	6	2	4	2	1	2	3	3	4	3
ERX2465463	2	4	3	3	1	3	3	4	3	2	4	2	3	2	5	2	5	2	2	1	3	4	2	3
ERX2465671	2	5	5	3	1	3	3	4	3	2	2	2	3	2	5	2	5	2	1	1	3	4	2	3
ERX2465505	2	4	4	3	1	3	2	4	3	2	4	2	4	2	4	2	5	2	2	1	3	4	2	3
ERX2465275	3	5	2	3	2	2	3	4	4	2	3	2	5	1	8	3	5	2	1	1	3	4	2	2
ERX2465563	2	5	1	4	3	3	4	4	2	2	2	2	3	2	6	2	6	2	2	1	3	4	2	3
ERX2465363	2	1	2	5	1	3	1	4	3	4	3	3	1	3	6	2	5	2	2	1	3	4	2	3
ERX2465161	2	5	3	5	3	3	2	3	3	4	1	3	6	3	5	2	5	2	2	1	3	4	2	3
ERX2465596	2	7	3	6	4	5	4	2	4	2	3	2	2	4	8	2	5	3	2	1	3	4	2	3
ERX2465487	2	7	3	5	4	5	5	2	4	2	3	4	2	4	8	2	5	3	2	1	3	4	2	3
ERX2465514	2	5	1	2	3	4	2	4	3	2	3	2	4	3	4	2	5	2	2	1	3	3	2	3
ERX2465580	2	7	3	4	3	5	4	2	4	2	3	4	2	4	7	2	5	3	2	1	3	4	2	3
ERX2465502	2	5	3	6	5	5	3	2	4	2	3	4	2	4	9	2	5	3	2	1	3	4	2	3
ERX2465619	6	2	2	4	3	4	1	4	4	2	2	1	8	12	7	2	6	2	2	2	3	3	6	3
ERX2465313	5	2	3	4	3	5	2	2	9	2	7	1	3	12	4	2	6	1	2	2	3	3	4	3
ERX2465409	2	1	4	7	3	4	4	2	4	2	2	4	2	3	5	2	5	3	2	1	3	4	2	3
ERX2465219	2	5	3	5	3	3	2	3	3	4	4	3	6	3	6	2	3	2	1	1	3	2	2	3
ERX2465266	2	8	3	5	5	5	4	2	4	4	3	4	2	4	3	2	5	3	2	1	3	4	2	3
ERX2465531	2	1	4	7	4	4	4	2	-2	2	2	4	-2	3	4	2	5	3	2	1	3	4	2	3
ERX2465397	2	1	4	7	4	4	4	2	4	2	2	4	2	3	5	2	5	3	2	1	3	4	2	3
ERX2465303	2	5	1	4	1	3	2	4	2	1	2	2	3	3	7	2	6	2	2	1	2	4	1	5

ERX2465245	2	5	3	6	4	5	3	2	4	2	3	4	2	4	7	2	5	3	2	1	3	4	2	3
ERX2465674	3	5	2	3	3	3	3	4	4	2	3	2	5	1	9	3	5	2	1	1	3	4	2	3
ERX2465509	2	5	1	2	3	3	2	4	4	2	2	2	3	3	5	2	5	2	1	1	3	4	2	3
ERX2465235	2	1	2	5	1	3	3	1	2	4	3	3	1	3	8	2	5	2	2	1	3	2	2	2
ERX2465300	2	4	4	4	3	3	2	3	3	4	3	3	6	3	8	2	5	2	2	1	3	4	2	3
ERX2465199	2	5	4	6	3	3	2	3	3	4	3	3	4	3	5	2	5	2	2	1	3	4	2	3
ERX2465551	2	5	2	3	2	3	2	4	3	2	3	2	2	2	5	2	5	2	2	1	3	4	2	3
ERX2465170	2	6	3	4	3	4	2	3	3	4	7	3	2	4	5	2	5	2	2	1	3	4	2	3
ERX2465622	2	1	4	7	4	4	4	2	4	2	2	4	2	3	5	2	5	4	2	1	3	4	2	3
ERX2465686	2	5	3	2	3	3	3	4	3	2	3	2	2	5	3	2	5	2	2	1	3	4	1	2
ERX2465260	3	5	2	2	3	3	2	5	3	4	2	1	2	3	5	2	4	2	2	2	3	3	3	3
ERX2465390	2	5	3	5	3	5	2	3	3	4	4	2	3	2	7	2	3	2	1	1	3	2	2	3
ERX2465458	2	6	3	5	3	3	2	3	3	3	3	3	4	2	7	2	3	2	2	1	3	2	2	3
ERX2465213	2	5	3	5	3	3	2	3	3	4	3	3	7	3	5	2	5	2	2	1	3	4	2	3
ERX2465529	2	6	3	5	3	3	2	3	3	3	3	3	4	2	7	2	3	2	2	1	3	2	2	3
ERX2465371	2	5	3	6	4	5	3	2	4	2	3	4	2	4	7	2	5	3	2	1	3	4	2	3
ERX2465506	2	7	3	6	4	7	3	2	4	2	3	2	2	5	8	2	5	3	2	1	3	4	2	3
ERX2465659	2	6	3	2	3	3	2	4	3	2	3	2	3	3	6	2	5	2	2	1	3	4	1	3
ERX2465520	2	5	3	6	4	5	3	2	4	2	3	4	2	4	7	2	5	3	2	1	3	4	2	3
ERX2465537	2	7	3	2	3	5	4	4	4	4	3	2	6	5	8	2	5	4	2	1	3	4	2	3
ERX2465259	2	7	1	3	3	3	2	4	3	2	6	2	5	2	5	1	6	2	2	1	3	4	2	3
ERX2465336	2	4	1	3	3	4	1	4	2	2	5	2	3	2	4	2	6	2	2	1	3	4	2	3
ERX2465457	2	4	5	3	1	3	2	2	2	2	15	2	3	2	5	2	5	2	2	1	3	4	2	3
ERX2465556	2	2	3	4	1	3	2	4	2	2	1	2	4	3	7	2	6	2	2	1	3	4	2	1
ERX2465365	2	4	2	3	4	3	2	3	2	2	3	2	4	2	3	2	5	2	2	1	3	4	2	3
ERX2465256	2	1	2	5	1	3	1	4	3	4	3	3	1	3	7	2	5	2	2	1	3	4	2	3
ERX2465683	2	4	4	3	1	2	2	5	3	2	5	2	4	2	5	2	5	2	2	1	3	4	2	3
ERX2465374	2	4	3	3	3	3	2	3	3	4	5,5	3	2	3	7	2	5	2	2	1	3	4	2	3
ERX2465345	2	4	1	3	1	3	2	4	3	2	5	2	4	2	5	2	6	2	2	1	3	4	2	3
ERX2465575	2	7	3	3	3	5	4	4	4	4	3	2	5	5	7	2	5	3	2	1	3	4	2	3
ERX2465247	2	4	1	6	4	2	1	4	3	4	3	3	-2	2	2	2	4	2	2	2	4	3	2	3
ERX2465632	2	7	3	3	3	5	4	4	4	2	3	4	6	4	5	2	5	2	2	1	3	4	2	3
ERX2465536	5	2	3	4	3	6	2	4	6	1	5	1	3	6	6	2	6	3	2	2	3	3	1	3
ERX2465585	2	8	4	4	3	4	4	4	2	2	4	2	3	2	5	2	5	2	2	1	2	4	2	3
ERX2465428	2	5	4	5	3	3	2	3	3	4	3	3	3	3	5,5	2	5	2	2	1	3	4	2	3
ERX2465455	2	5	2	4	3	3	2	4	3	4	3	3	3	4	10	2	5	2	2	1	3	4	2	3
ERX2465205	2	7	3	5	4	8	4	2	4	2	3	4	2	4	8	2	5	4	2	1	3	3	2	3
ERX2465600	2	5	3	4	3	3	2	3	3	4	3	3	3	2	7	2	5	2	1	1	3	4	2	3
ERX2465444	2	8	4	4	3	4	4	5	2	2	6	2	2	2	5	2	4	2	2	1	2	4	2	3
ERX2465340	2	4	3	4	3	3	2	4	2	1	4	2	2	3	7	2	6	2	2	1	3	4	2	3
ERX2465582	3	5	4	5	3	3	2	3	3	4	3	3	5	3	6	2	5	2	2	1	3	4	2	3
ERX2465401	2	1	1	3	3	3	2	4	2	2	2	2	5	2	2	2	6	2	2	1	3	4	2	3
ERX2465450	2	5	1	3	1	3	3	4	3	1	2	2	5	3	7	2	6	2	2	1	2	4	1	5
ERX2465443	2	5	3	6	4	5	3	2	4	2	3	4	2	4	7	2	5	3	2	1	3	4	2	3
ERX2465528	3	4	3	4	3	3	4	4	4	2	3	3	4	1	9	2	5	2	2	1	3	4	2	3
ERX2465431	2	5	5	5	3	3	2	3	3	4	5	1	6	4	5	2	5	2	2	1	3	4	2	3

ERX2465367	2	5	3	5	3	3	2	3	3	4	3	3	3	3	5	2	5	2	2	1	3	4	2	2
ERX2465251	2	5	3	6	4	5	3	2	4	2	3	4	2	3	7	2	3	3	2	1	3	4	2	3
ERX2465479	2	5	3	3	2	3	2	4	3	2	3	2	2	2	5	2	2	2	2	1	3	2	2	3
ERX2465513	5	2	3	6	3	5	2	2	9	2	8	1	4	7	3	2	7	1	2	2	3	3	4	3
ERX2465377	2	5	3	5	3	3	2	3	3	4	2	3	4	3	4	2	5	2	2	1	3	4	2	3
ERX2465687	2	3	3	3	3	5	4	4	4	2	3	5	7	4	7,<br>5	2	5	3	2	1	3	4	2	3
ERX2465642	1	5	3	3	3	4	2	5	4	2	3	2	2	3	4	2	5	2	1	1	3	4	1	3
ERX2465273	2	5	3	6	4	5	3	2	4	2	3	4	2	4	7	2	5	3	2	1	3	4	2	2
ERX2465578	2	5	4	2	1	2	3	4	3	2	5	2	3	2	3	2	6	2	2	1	3	4	2	3
ERX2465524	2	5	3	3	1	2	2	4	3	2	5	2	3	2	3	2	5	2	2	1	3	4	2	3
ERX2465375	2	5	3	3	1	3	2	4	3	2	7	2	3	2	5	2	5	2	2	1	3	4	2	3
ERX2465201	2	8	3	5	3	5	4	2	4	2	3	4	2	4	8	2	7	3	2	1	3	4	2	3
ERX2465562	2	5	3	5	3	3	2	3	3	4	3	2	5	2	7	2	3	2	1	1	3	2	2	3
ERX2465301	2	1	2	5	1	3	1	4	3	4	3	3	1	3	7	2	5	2	2	1	3	4	2	3
ERX2465685	2	4	3	6	4	3	4	2	4	2	3	4	2	6	6	2	5	3	2	1	3	4	2	3
ERX2465386	2	3	3	3	3	3	2	4	3	2	3	2	4	2	5	1	5	2	1	1	3	4	2	3
ERX2465196	2	6	3	5	3	3	2	3	3	3	3	3	4	2	7	2	3	2	2	1	3	2	2	3
ERX2465226	2	7	4	4	3	4	2	4	2	2	3	2	3	2	5	2	6	2	2	1	3	4	2	3
ERX2465480	2	6	5	3	1	4	2	4	2	2	3	2	2	2	6	2	5	2	2	1	3	4	2	3
ERX2465388	3	5	2	2	3	3	2	3	5	4	2	1	2	3	5	2	4	2	2	2	3	3	4	1
ERX2465643	3	6	4	3	3	3	3	4	3	2	3	2	2	1	1<br>0	2	6	2	2	1	3	4	2	3
ERX2465554	4	2	3	4	3	4	2	4	-<br>2	1	4	1	-2	7	4	2	7	3	2	2	3	3	1	3
ERX2465250	2	5	3	4	3	3	2	3	3	4	1	3	4	3	7	2	5	2	2	1	3	4	2	2
ERX2465280	2	2	3	5	2	3	2	3	4	4	3	3	3	3	6	2	5	2	2	1	3	4	2	3
ERX2465620	2	1	2	5	1	3	1	4	2	4	3	3	1	3	7	2	5	2	2	1	3	4	2	3
ERX2465570	4	5	4	3	3	3	2	4	3	2	3	2	3	2	3	2	6	2	2	1	3	4	2	3
ERX2465311	2	2	3	5	2	3	2	3	4	4	3	3	3	3	6	2	5	2	2	1	3	4	2	3
ERX2465321	3	2	2	4	3	4	2	4	4	2	2	1	8	4	7	2	6	2	2	2	3	3	6	3
ERX2465510	2	5	3	5	3	3	1	3	3	4	3	3	6	3	7	2	3	2	2	1	3	2	2	3
ERX2465238	2	5	3	5	3	3	2	3	3	4	3	3	5	3	5	2	5	2	2	1	3	4	2	3
ERX2465329	2	7	3	6	4	5	2	2	4	2	3	1	2	4	8	2	5	3	2	1	3	4	2	3
ERX2465415	2	4	2	4	3	3	3	4	2	1	2	3	2	3	9	1	6	2	2	1	3	4	2	3
ERX2465189	2	5	2	3	3	3	1	4	2	2	3	2	4	2	5	2	5	2	2	1	3	4	2	3
ERX2465616	7	2	2	4	2	4	1	4	3	2	2	1	9	9	7	2	6	3	2	2	3	3	4	3
ERX2465590	2	6	3	4	3	3	2	3	3	4	3	3	3	3	5	2	3	2	2	1	3	2	2	3
ERX2465414	2	5	3	6	4	5	3	2	4	2	3	4	2	4	7	2	5	3	2	1	3	4	2	3
ERX2465518	2	5	3	6	3	3	2	3	3	4	3	3	5	3	5	2	5	2	2	1	3	4	2	3
ERX2465446	9	2	3	4	2	5	2	4	7	2	4	1	2	5	6	3	5	3	2	2	3	3	4	3
ERX2465416	2	5	3	3	3	3	2	4	4	2	1<br>9	2	5	3	5	2	5	2	1	1	3	4	2	3
ERX2465439	2	6	1	4	2	2	4	4	1	1	2	2	2	3	4	2	6	2	2	1	3	4	2	2
ERX2465159	2	5	3	4	2	3	4	2	2	2	1	2	3	3	6	2	6	2	2	1	2	4	2	1
ERX2465654	2	4	4	3	1	2	2	5	3	2	5	2	4	2	5	2	5	2	2	1	3	4	2	3
ERX2465484	2	7	3	5	4	5	5	2	4	2	3	4	2	4	8	2	5	3	2	1	3	4	2	3
ERX2465344	2	5	1	3	3	3	2	4	4	2	-<br>2	2	6	3	5	2	5	2	1	1	3	4	2	3
ERX2465666	2	1	4	7	4	4	4	2	4	2	2	4	2	3	5	2	5	3	2	1	3	4	2	3

ERX2465364	2	7	3	3	3	5	4	4	4	4	3	2	6	5	8	2	5	3	2	1	3	4	2	3
ERX2465281	2	4	3	4	2	3	2	3	3	4	3	3	2	3	7	2	5	2	2	1	3	4	2	3
ERX2465566	2	4	3	6	4	5	3	2	4	2	3	4	2	4	7	2	5	3	2	1	3	4	2	3
ERX2465612	2	1	4	7	4	4	4	2	4	2	2	4	2	3	5	2	5	3	2	1	3	4	2	3
ERX2465667	2	5	1	5	3	3	2	3	3	4	1	3	4	3	7	2	6	2	2	1	3	4	2	3
ERX2465681	2	1	4	7	4	4	5	2	3	2	2	1	2	3	5	2	5	3	1	1	2	4	2	2
ERX2465285	6	2	3	4	2	5	2	4	6	2	3	1	2	5	6	3	5	3	2	2	3	3	4	3
ERX2465350	2	8	3	5	6	5	4	2	4	4	3	4	2	4	3	2	5	3	2	1	3	4	2	3
ERX2465241	2	5	3	6	5	5	3	2	4	2	3	4	2	4	7	2	5	3	2	1	3	4	2	3
ERX2465592	5	2	3	4	3	5	2	4	8	2	8	1	2	7	4	2	5	3	2	2	1	3	3	3
ERX2465611	2	1	2	5	1	3	1	4	3	4	3	3	2	3	9	2	5	2	1	1	3	4	2	3
ERX2465532	3	5	2	5	3	3	2	3	3	4	3	3	5	3	6	2	5	2	2	1	3	4	2	3
ERX2465166	2	7	3	5	4	5	5	2	4	2	3	4	2	6	8	2	5	3	2	1	3	4	2	3
ERX2465404	2	7	3	6	4	4	4	2	4	4	4	4	2	4	7	2	5	2	2	1	3	4	2	3
ERX2465433	2	5	1	5	2	3	4	3	3	6	3	3	2	3	2	2	1	2	1	1	3	4	2	2
ERX2465432	2	4	4	7	4	6	1	2	4	2	3	4	2	4	7	2	5	3	2	1	3	4	2	3
ERX2465356	2	5	2	3	4	3	2	4	2	2	3	2	2	2	5	2	5	2	2	1	3	4	2	3
ERX2465214	2	8	3	5	5	5	4	2	4	4	3	4	2	4	3	2	5	3	2	1	3	4	2	3
ERX2465218	2	7	3	3	3	5	4	4	4	3	3	2	3	5	8	2	5	3	2	1	3	4	2	3
ERX2465451	2	5	3	5	3	2	2	3	3	4	3	3	4	3	7	2	3	2	2	1	3	2	2	3
ERX2465284	2	1	2	5	1	3	1	4	3	4	3	3	2	3	8	2	5	2	2	1	3	4	2	3
ERX2465225	2	2	4	4	2	2	3	2	2	1	2	2	2	3	4	1	3	2	2	1	3	4	2	3
ERX2465419	2	5	4	3	1	2	2	4	2	2	3	2	4	2	4	2	5	2	2	1	2	4	2	3
ERX2465606	2	5	4	3	3	3	2	4	3	2	3	2	3	2	5	2	6	2	2	1	3	4	2	3
ERX2465328	2	2	4	3	1	3	2	4	3	2	2	2	3	2	5	2	5	2	2	1	3	4	2	3
ERX2465488	2	6	3	1	3	5	4	4	3	4	3	4	6	4	8	2	5	3	2	1	3	4	2	3
ERX2465639	3	6	2	2	3	3	2	4	4	2	1	2	5	1	5	2	5	2	2	1	2	4	2	3
ERX2465183	2	5	2	4	3	2	1	4	2	2	3	2	4	2	3	2	5	2	2	1	2	5	2	3
ERX2465478	2	5	2	3	3	3	1	4	4	2	4	2	2	3	3	2	3	2	2	1	3	4	2	2
ERX2465464	3	5	2	2	3	3	2	5	5	4	3	1	2	2	5	2	4	2	2	2	3	3	3	3
ERX2465644	2	4	1	4	1	3	4	4	2	1	2	2	3	3	7	2	6	2	2	1	2	2	1	5
ERX2465202	2	3	4	4	3	3	4	4	2	2	1	2	2	2	6	2	1	2	2	1	3	4	2	1
ERX2465212	2	1	2	5	1	3	1	4	3	6	3	3	1	3	7	2	5	2	2	1	3	4	2	3
ERX2465670	2	4	3	2	3	3	2	4	3	2	3	2	2	5	7	2	5	2	2	1	3	2	1	2
ERX2465406	2	5	5	4	3	3	3	4	3	2	3	2	3	2	3	2	5	2	2	1	3	4	2	3
ERX2465299	2	5	2	3	2	3	1	5	4	2	2	2	2	3	4	2	5	2	2	1	3	4	2	3
ERX2465501	3	5	2	2	3	2	2	3	6	4	2	1	2	3	5	2	4	2	2	2	3	3	5	3
ERX2465511	5	2	3	4	3	2	2	3	9	2	4	1	6	3	5	2	6	3	2	2	3	3	3	3
ERX2465157	2	6	3	4	3	3	2	3	3	4	3	3	3	3	5	2	3	2	2	1	3	2	2	3
ERX2465519	2	5	3	6	4	5	3	2	4	2	3	4	2	4	7	2	5	3	2	1	3	4	2	3
ERX2465232	2	5	3	6	4	5	3	2	4	2	3	4	2	4	7	2	5	3	2	1	3	4	2	3
ERX2465663	5	2	2	5	3	4	1	4	4	2	2	1	8	10	7	2	6	3	2	2	3	3	6	3
ERX2465167	2	4	3	3	3	3	2	3	2	4	3	4	2	3	7	2	5	2	2	1	3	4	2	3
ERX2465462	2	5	4	5	3	3	2	3	3	4	3	3	3	3	5,5	2	5	2	2	1	3	4	2	3
ERX2465627	2	4	3	3	3	3	2	3	3	4	5,5	3	2	3	7	2	5	2	2	1	3	4	2	3
ERX2465308	2	5	2	5	4	5	3	2	4	2	4	4	2	4	7	2	5	3	2	1	3	4	2	3

ERX2465661	2	5	3	5	3	3	2	4	3	4	3	3	4	3	6	2	3	2	1	1	3	2	2	3
ERX2465626	3	5	2	2	3	3	2	5	3	4	2	1	4	3	5	2	4	2	2	2	3	3	5	3
ERX2465581	2	5	3	6	4	5	3	2	4	2	6	4	2	4	7	2	5	3	2	1	3	4	2	3
ERX2465655	4	2	2	4	2	6	2	4	7	2	2	1	5	4	7	2	6	2	2	2	3	3	6,5	3
ERX2465500	2	4	4	3	1	2	2	5	3	2	5	2	4	2	5	2	5	2	2	1	3	4	2	3
ERX2465237	2	7	3	4	4	5	5	2	4	2	3	2	2	4	8	2	5	3	2	1	3	4	2	3
ERX2465669	2	5	4	5	3	3	2	3	3	4	2	3	6	3	7	2	3	2	2	1	3	2	2	3
ERX2465517	2	6	1	3	3	3	3	3	3	4	2	3	4	2	6	2	5	2	2	1	3	4	3	3
ERX2465605	4	2	3	4	4	5	2	4	8	2	3	1	8	6	5	2	8	3	2	2	3	3	4	3
ERX2465447	2	7	3	3	3	5	4	4	4	4	3	2	6	5	8	2	5	4	2	1	3	4	2	3
ERX2465460	5	2	2	4	3	4	1	4	4	2	2	1	8	7	7	2	6	3	2	2	3	3	6,5	3
ERX2465267	2	5	4	3	3	3	2	4	3	2	3	2	2	2	5	2	6	2	2	1	3	4	2	3
ERX2465521	2	4	3	3	3	3	2	4	4	2	-2	2	4	3	5	2	5	2	1	1	3	4	2	2
ERX2465650	2	3	3	6	4	5	3	2	3	2	3	4	2	4	7	2	5	3	2	1	3	4	2	3
ERX2465651	2	5	3	5	3	3	3	3	3	4	3	3	5	3	6	2	5	2	2	1	3	5	2	3
ERX2465378	2	7	3	6	4	5	4	2	4	2	3	4	2	4	8	2	5	3	2	1	3	4	2	3
ERX2465191	2	7	3	6	4	5	4	2	4	2	3	4	2	4	8	1	5	3	2	1	3	4	2	3
ERX2465630	2	5	6	4	3	3	3	4	2	1	2	1	2	2	3	1	6	2	2	1	3	4	2	3
ERX2465630	2	7	6	4	3	3	3	4	2	1	2	1	2	2	3	1	6	2	2	1	3	4	2	3
ERX2465469	2	1	2	10	2	2	2	4	4	4	3	2	2	3	7	2	1	2	2	1	3	4	2	3
ERX2465574	0	5	3	3	3	3	2	4	3	2	3	2	3	2	5	2	6	2	2	1	3	4	2	3
ERX2465352	2	5	1	3	3	3	2	4	3	2	5	2	2	2	4	2	6	2	2	1	3	4	2	3
ERX2465257	2	5	2	3	1	3	2	4	3	2	2	2	2	2	5	2	5	2	2	1	3	4	2	3
ERX2465569	2	1	2	5	1	3	1	4	3	5	3	3	1	3	7	2	5	2	2	1	3	4	2	3
ERX2465298	2	5	3	6	4	5	3	2	4	2	3	4	2	4	7	2	5	3	2	1	3	4	2	3
ERX2465545	2	5	3	5	3	3	2	3	2	4	3	3	5	3	7	2	6	2	2	1	3	4	2	3
ERX2465679	2	1	2	5	1	3	1	4	3	4	3	3	2	3	5	2	5	2	2	1	3	4	2	3
ERX2465682	2	5	2	3	3	2	3	4	2	1	2	2	2	3	7	1	6	2	2	1	3	4	2	4
ERX2465515	2	7	3	5	4	5	5	2	3	2	3	4	2	4	8	2	5	3	2	1	3	4	2	3
ERX2465657	2	5	3	6	3	5	4	2	4	2	4	4	2	4	2	2	5	3	2	1	3	4	2	3
ERX2465272	2	4	1	2	4	3	2	4	3	2	3	2	3	3	5	2	5	2	1	1	3	3	2	3
ERX2465602	2	7	3	4	3	5	4	2	4	2	3	4	2	4	7	2	5	3	2	1	3	4	2	3
ERX2465535	2	5	3	5	3	3	2	4	3	4	3	3	4	3	6	2	3	2	1	1	3	2	2	3
ERX2465452	2	5	3	7	4	6	1	2	4	2	3	4	2	4	7	2	5	3	2	1	3	4	2	3
ERX2465617	2	7	3	3	3	3	4	4	3	4	3	4	6	4	7	2	5	3	2	1	3	4	2	3
ERX2465690	2	5	3	5	3	3	2	3	3	4	3	3	3	3	5	2	6	2	2	1	3	4	2	3
ERX2465382	2	5	4	4	2	3	4	2	2	2	1	2	3	2	6	2	6	1	2	1	3	4	2	1
ERX2465351	2	5	3	6	4	5	3	2	4	2	3	4	2	4	7	2	5	3	2	1	3	4	2	3
ERX2465274	2	5	2	5	4	3	2	3	3	4	3	1	3	3	5	2	5	2	2	1	3	4	2	3
ERX2465316	2	5	3	7	4	5	3	2	4	2	2	4	2	4	7	2	5	3	2	1	3	4	2	3
ERX2465217	2	1	4	7	4	4	4	2	4	2	2	4	2	3	5	2	5	3	2	1	3	4	2	3
ERX2465249	2	5	6	4	3	3	4	4	2	2	5	2	2	4	8	2	5	2	2	1	3	4	2	3
ERX2465290	2	5	3	5	3	3	2	3	3	4	3	3	5	3	7	2	3	2	2	1	3	2	2	3
ERX2465164	2	1	4	7	4	4	4	2	4	2	2	4	2	3	5	2	5	3	2	1	3	4	2	3
ERX2465403	5	2	3	3	5	4	2	4	9	2	6	1	2	2	5	2	5	3	2	2	3	3	4	3
ERX2465348	5	2	2	4	2	5	2	4	7	2	2	1	7	9	8	2	6	2	2	2	3	3	5	3

ERX2465248	2	5	4	3	1	3	2	4	3	2	1	2	2	2	4	2	5	2	2	1	3	4	2	3
ERX2465224	1	1	2	5	1	3	4	4	3	4	3	3	2	3	7	2	5	2	2	1	3	4	2	3
ERX2465240	5	2	2	4	3	4	1	4	4	2	2	1	1	10	8	2	6	3	2	2	3	3	6	3
ERX2465542	2	5	1	4	3	3	4	4	2	2	2	2	3	2	6	2	6	2	2	1	3	4	2	3
ERX2465402	2	5	3	6	4	5	3	2	4	2	3	4	2	4	7	2	5	3	2	1	3	4	2	3
ERX2465193	2	5	4	5	3	3	2	3	3	3	4	3	6	3	7	2	3	2	2	1	3	2	2	3
ERX2465370	1,5	5	6	4	3	3	4	3	3	6	3	1	4	4	8	2	5	2	2	1	3	4	2	3
ERX2465467	2	5	3	3	2	5	4	4	4	4	3	2	6	4	8	2	5	3	2	1	1	4	2	3
ERX2465558	2	5	3	3	3	4	2	3	4	2	5	2	2	3	9	2	5	2	2	1	3	4	2	3
ERX2465656	2	8	2	4	3	4	4	4	2	2	5	2	3	2	5	2	4	2	2	1	3	4	2	3
ERX2465381	2	7	3	3	3	5	4	4	4	4	3	2	5	5	8	2	5	3	2	1	3	4	2	3
ERX2465561	2	1	2	5	1	3	1	2	3	4	3	3	2	3	8	2	5	2	2	1	3	4	2	3
ERX2465295	2	3	6	4	3	3	3	4	2	1	2	3	2	2	8	1	6	2	2	1	3	4	2	3
ERX2465459	3	5	4	5	3	2	2	3	3	4	4	3	4	3	7	2	5	2	1	1	3	2	2	3
ERX2465342	2	5	3	6	4	6	1	2	4	2	3	4	2	3	4	2	5	3	2	1	3	4	2	3
ERX2465613	2	1	2	7	2	2	3	4	3	4	3	3	2	3	3	2	5	2	2	1	3	4	2	3
ERX2465243	2	7	3	6	4	4	4	2	4	4	4	4	2	3	7	2	5	2	1	1	3	4	2	3
ERX2465421	2	5	3	6	4	5	3	2	4	2	3	4	2	4	7	2	5	3	2	1	3	4	2	3
ERX2465252	2	7	1	3	3	5	2	4	4	4	3	2	6	6	8	2	5	3	2	1	3	4	2	3
ERX2465265	2	5	3	5	3	2	3	3	3	4	3	3	2	3	6	2	6	2	2	1	3	4	2	3
ERX2465499	2	5	3	6	4	5	4	2	4	2	3	4	2	4	7	2	5	3	2	1	3	4	2	3
ERX2465354	2	1	2	5	1	3	1	4	3	4	3	3	1	3	8	2	5	2	2	1	3	4	2	3
ERX2465423	5	2	2	4	3	4	1	4	4	2	2	1	8	10	7	2	6	3	2	2	3	3	4	3
ERX2465297	2	5	3	6	3	5	3	2	2	2	5	4	2	4	7	2	5	3	2	1	3	4	2	3
ERX2465680	5	2	2	4	2	6	1	4	7	2	2	1	5	4	7	2	6	2	2	2	3	3	6	3
ERX2465385	3	5	5	3	2	3	1	4	3	2	3	2	4	1	6	2	5	2	2	1	3	4	2	2
ERX2465527	2	5	2	4	3	2	3	4	2	1	2	2	2	3	6	1	6	2	2	1	3	4	2	3
ERX2465182	2	5	1	3	1	4	5	4	2	1	2	2	2	3	7	2	8	2	2	1	2	4	1	5
ERX2465179	2	5	4	3	3	3	2	4	3	2	3	2	3	2	5	2	6	2	2	1	3	4	2	3
ERX2465425	2	5	2	3	4	2	2	4	2	2	4	2	4	2	2	2	5	2	2	1	3	4	2	3
ERX2465271	2	7	3	4	3	4	4	2	3	2	3	2	2	4	7	2	5	3	2	1	3	4	2	2
ERX2465493	2	5	3	4	3	3	4	4	2	2	1	2	4	3	6	2	6	2	2	1	2	4	2	1
ERX2465277	2	1	4	7	4	4	4	2	4	2	2	4	2	3	5	2	5	3	2	1	3	4	2	3
ERX2465332	2	7	2	6	4	5	8	2	3	2	3	4	2	4	8	2	5	3	2	1	3	4	2	3
ERX2465426	1	5	3	3	3	3	1	4	3	2	3	2	2	3	1	2	5	2	1	1	2	3	2	3
ERX2465599	2	7	3	6	4	4	4	2	2	4	4	4	2	4	7	2	5	2	2	1	3	4	2	3
ERX2465560	2	7	3	3	3	5	4	4	4	4	3	2	6	5	7	2	5	3	2	1	3	4	2	3
ERX2465353	2	3	3	6	4	5	3	2	3	2	3	4	2	4	7	2	5	3	2	1	3	4	2	3
ERX2465172	2	1	2	6	2	2	3	4	3	4	3	3	2	3	8	2	5	2	2	1	3	4	2	3
ERX2465483	2	4	2	3	3	3	1	5	3	2	4	2	3	3	3	2	5	1	2	1	3	4	2	2
ERX2465494	2	5	4	5	3	3	2	3	3	4	3	3	7	3	7	2	6	2	2	1	3	4	2	3
ERX2465325	2	5	3	3	3	5	4	4	4	4	3	2	6	5	8	2	5	3	2	1	3	4	2	3
ERX2465291	2	4	2	3	4	3	2	4	2	2	3	2	4	2	5	2	5	2	2	1	1	4	2	3
ERX2465322	2	4	4	6	4	6	1	2	4	2	3	4	2	6	7	2	5	3	2	1	3	4	2	3
ERX2465552	2	4	4	3	3	3	2	4	3	2	2	2	3	2	3	2	5	2	2	1	3	4	2	3
ERX2465178	2	1	4	7	4	3	4	2	4	2	2	4	2	3	5	2	5	3	2	1	3	4	2	3

ERX2465598	2	5	3	5	3	3	2	3	3	4	3	3	5	3	5	2	5	2	2	1	3	4	2	3
ERX2465435	2	5	3	5	2	3	2	3	3	4	4	3	6	4	7	2	5	2	2	1	3	4	2	3
ERX2465604	2	7	3	3	3	5	4	4	3	2	3	2	5	6	8	2	5	3	2	1	3	4	2	3
ERX2465475	5	2	2	4	3	4	1	4	4	2	2	1	8	10	7	2	6	3	2	2	3	3	6	3
ERX2465658	3	6	4	3	3	3	3	4	3	2	3	2	2	1	10	2	6	2	2	1	3	4	2	3
ERX2465376	2	5	3	5	3	3	2	3	3	4	4	1	4	4	7	2	5	2	1	1	3	4	2	2
ERX2465279	2	5	3	5	3	2	2	3	3	4	3	3	4	3	7	2	3	2	2	1	3	2	2	3
ERX2465586	2	7	4	3	3	5	4	4	3	2	3	2	5	5	8	2	5	3	2	1	3	4	2	3
ERX2465546	2	7	3	3	3	5	4	4	4	4	3	2	6	5	8	2	6	3	2	1	3	4	2	3
ERX2465676	2	5	2	5	4	4	2	3	3	4	3	3	3	3	5	2	5	2	2	1	3	4	2	3
ERX2465564	5	2	2	4	3	4	2	2	9	2	5	1	8	11	4	2	6	1	2	2	3	3	4	3
ERX2465541	2	5	1	4	1	2	2	4	2	1	2	2	3	3	7	2	7	2	2	1	2	4	1	5
ERX2465195	5	2	3	4	3	5	2	4	9	2	4	1	6	5	6	2	3	3	2	2	3	3	4	3
ERX2465618	2	1	4	7	4	4	4	2	4	2	2	4	2	3	5	2	5	3	2	1	3	4	2	3
ERX2465230	2	4	4	3	3	3	2	4	3	2	3	2	5	2	3	2	5	2	2	1	3	4	2	3
ERX2465434	3	5	2	3	3	3	3	4	4	2	3	2	5	1	9	3	5	2	1	1	3	4	2	3
ERX2465634	3	5	2	2	3	3	2	5	7	4	2	1	4	3	4	2	4	2	2	2	3	3	4	3
ERX2465565	2	1	2	4	1	3	1	4	3	4	3	3	1	3	7	2	5	2	1	1	3	4	2	3
ERX2465398	2	1	2	5	1	3	1	4	3	5	3	3	1	3	7	2	5	2	2	1	3	4	2	3
ERX2465417	7	2	3	4	2	5	2	4	4	2	4	1	2	5	6	2	5	3	2	2	3	3	4	3
ERX2465287	2	7	3	5	4	6	5	2	4	2	3	4	2	4	7	2	5	3	2	1	3	4	2	3
ERX2465496	2	1	2	5	1	3	1	4	3	4	3	3	1	3	7	2	5	2	2	1	3	4	2	3
ERX2465197	2	4	1	6	5	4	3	2	4	2	3	2	2	4	8	2	5	3	2	1	3	4	2	3
ERX2465491	2	1	4	7	4	4	4	2	4	2	2	4	2	3	5	2	5	3	2	1	3	4	2	3
ERX2465269	2	6	1	4	2	3	4	4	2	1	2	2	2	3	3	2	6	2	2	1	3	4	1	5
ERX2465572	5	2	4	4	3	4	2	4	5	1	4	1	3	6	6	2	6	3	2	2	3	3	1	3
ERX2465629	2	4	3	4	3	3	4	4	2	2	1	2	3	3	4	2	7	2	2	1	3	4	2	1
ERX2465436	3	5	2	2	3	3	2	5	6	4	2	1	2	3	2	2	4	2	2	2	2	3	4	3
ERX2465233	2	5	4	3	3	3	2	4	3	2	3	2	3	2	5	2	6	2	2	1	3	4	2	3
ERX2465175	2	5	3	5	3	2	2	3	3	4	3	2	5	2	6	2	5	2	2	1	3	4	2	3
ERX2465607	2	7	3	2	3	5	4	4	4	4	3	2	6	5	6	2	5	4	2	1	3	4	2	3
ERX2465306	2	5	3	6	4	5	3	2	4	2	3	4	2	4	7	2	5	3	2	1	3	4	2	3
ERX2465320	2	5	3	5	2	3	2	3	2	4	3	3	5	3	6	2	5	2	2	1	3	4	2	3
ERX2465320	2	5	2	5	2	3	2	3	2	4	3	3	5	3	6	2	5	2	2	1	3	4	2	3
ERX2465472	2	5	3	6	4	6	1	2	4	2	3	4	2	4	7	2	5	3	2	1	3	4	2	3
ERX2465310	3	5	4	3	3	3	3	4	4	2	3	2	2	1	11	2	6	2	2	1	3	4	2	3
ERX2465668	2	7	3	4	3	5	4	2	4	2	3	4	2	4	7	2	5	3	2	1	3	4	2	3
ERX2465430	2	6	3	5	3	3	2	3	3	3	3	3	4	2	7	2	3	2	2	1	3	2	2	3
ERX2465387	2	5	3	6	4	6	1	2	4	2	3	4	2	4	7	2	5	1	2	1	3	4	2	3
ERX2465156	7	2	4	4	3	5	2	4	7	2	4	1	11	6	6	2	6	3	1	2	3	3	5	3
ERX2465333	5	2	2	4	2	6	2	4	7	2	2	1	5	4	7	2	6	2	2	2	3	3	6	3
ERX2465442	2	5	3	5	3	3	2	3	3	4	3	3	6	2	5	2	5	2	2	1	4	4	2	3
ERX2465609	2	5	2	1	3	3	2	4	3	2	3	2	2	3	6	2	5	2	2	1	3	4	3	3
ERX2465468	2	5	2	6	4	5	3	2	4	2	3	4	2	4	8	2	5	3	2	1	3	4	2	3
ERX2465174	2	1	3	7	2	2	3	5	4	4	3	3	2	3	5	2	5	2	2	1	3	4	2	3

ERX2465326	2	5	3	6	3	3	2	3	3	4	3	1	4	3	6	1	5	2	2	1	3	4	2	3
ERX2465544	2	7	3	2	3	5	4	4	4	4	3	2	5	5	6	2	5	4	2	1	3	4	2	3
ERX2465368	2	5	3	5	2	3	2	3	3	4	3	3	6	3	6	2	5	2	2	1	3	4	2	3
ERX2465347	2	5	2	4	3	3	1	4	4	2	4	2	2	3	3	2	3	2	2	1	3	4	2	2
ERX2465220	2	7	3	5	4	6	5	2	4	2	3	4	2	3	8	2	5	3	2	1	3	4	2	3
ERX2465181	2	1	2	5	1	3	1	4	3	4	3	3	2	3	9	2	5	2	1	1	3	4	2	3
ERX2465158	2	3	3	2	4	3	2	4	3	2	4	2	2	3	2	2	5	2	2	1	3	4	1	3
ERX2465207	2	5	3	5	3	3	2	3	3	4	1	3	6	3	6	2	5	2	2	1	3	4	2	3
ERX2465379	2	5	3	3	3	5	4	4	4	4	3	2	6	5	8	2	5	3	2	1	3	4	2	3
ERX2465358	2	5	4	3	2	3	2	3	3	2	2	2	2	2	4	2	5	2	2	1	3	4	2	3
ERX2465530	2	5	2	5	3	3	2	3	3	4	3	2	4	2	5	2	3	2	1	1	3	2	2	3
ERX2465366	2	6	2	7	3	4	2	3	3	4	7	3	2	6	7	2	5	2	2	1	3	4	2	2
ERX2465497	2	5	4	4	3	3	4	3	3	2	3	3	3	3	10	2	5	1	2	1	3	4	1	3
ERX2465429	6	2	3	4	2	5	2	4	9	2	2	1	11	9	5	2	6	1	2	2	3	3	2	3
ERX2465305	2	7	3	3	3	4	4	4	4	4	3	2	5	5	8	2	5	3	2	1	3	4	2	3
ERX2465341	3	5	2	2	3	3	2	5	3	4	2	1	4	3	4	2	4	2	2	2	3	3	6	3
ERX2465163	2	4	3	4	2	3	2	4	2	2	1	2	4	3	4	2	6	2	1	1	3	4	2	1
ERX2465538	2	1	4	8	3	4	4	2	4	2	2	4	2	3	5	2	5	3	2	1	3	4	2	3
ERX2465649	6	2	4	4	2	5	2	4	7	2	3	1	2	5	6	2	5	3	2	2	3	3	5	3
ERX2465383	2	5	3	5	3	3	2	3	3	4	1	3	6	3	6	2	5	2	2	1	3	4	2	3
ERX2465482	2	1	2	5	1	3	1	4	3	4	3	2	1	3	10	2	5	2	2	1	3	4	2	3
ERX2465393	2	5	4	3	1	3	2	4	3	2	4	2	3	2	3	2	5	2	2	1	3	3	2	3
ERX2465165	2	4	4	4	3	3	4	4	2	2	3	2	3	2	5	2	4	2	2	1	3	4	2	3
ERX2465637	2	5	5	3	4	3	2	4	2	2	4	2	5	2	5	2	5	2	2	1	3	4	2	3
ERX2465261	2	4	1	4	2	3	2	4	2	1	2	2	3	3	3	2	6	2	2	1	3	4	1	5
ERX2465489	2	8	4	3	4	5	4	2	4	2	3	4	2	4	7	2	5	3	2	1	3	4	2	3
ERX2465456	2	7	3	2	4	5	4	2	4	2	2	5	2	4	8	2	5	3	2	1	3	4	2	3
ERX2465410	2	6	4	4	3	2	3	2	2	1	2	2	2	3	6	1	6	2	2	1	3	4	2	3
ERX2465407	2	7	3	6	4	5	4	2	-2	2	3	3	-2	4	8	2	5	3	2	1	3	4	2	3
ERX2465636	2	6	2	7	3	4	2	3	3	4	7	3	2	6	7	2	5	2	2	1	3	4	2	2
ERX2465595	2	5	3	4	3	2	4	4	1	2	6	2	3	3	6	2	6	2	2	1	3	3	2	3
ERX2465550	2	7	3	3	3	5	4	4	-2	2	3	2	5	5	8	2	5	3	2	1	3	4	2	3
ERX2465638	2	4	2	3	1	3	2	4	2	2	4	2	4	2	5	2	5	2	2	1	3	4	2	3
ERX2465445	2	5	3	4	3	2	2	3	3	4	3	3	3	4	7	2	3	2	2	1	3	2	2	3
ERX2465665	2	7	3	3	3	5	4	4	4	4	3	2	6	5	8	2	5	3	2	1	3	4	2	3
ERX2465294	5	2	3	4	3	5	2	2	8	2	5	1	6	10	4	2	6	1	2	2	3	3	4	3
ERX2465677	2	4	3	6	4	5	3	2	4	2	3	2	2	4	7	2	3	3	2	1	2	4	2	3
ERX2465369	2	5	3	3	3	3	2	4	2	2	3	2	2	2	6	2	5	2	2	1	3	4	2	3
ERX2465490	2	5	4	2	1	3	2	4	2	2	4	2	3	2	5	2	5	2	2	1	3	4	2	3
ERX2465173	2	8	2	4	3	4	4	4	2	2	7	2	3	2	5	2	4	2	2	1	3	4	2	3
ERX2465318	2	3	3	6	4	5	3	2	4	2	3	4	2	4	8	2	5	3	2	1	3	4	2	3
ERX2465359	2	1	2	5	1	2	4	4	2	4	3	1	1	3	7	2	5	2	2	1	2	4	2	3
ERX2465246	2	5	2	3	4	3	2	4	2	2	3	2	3	2	5	2	5	2	2	1	3	4	2	3
ERX2465355	2	4	2	3	3	3	2	2	2	1	2	2	2	3	6	1	6	2	2	1	3	4	2	3

ERX2465645	2	5	3	6	4	5	3	2	4	2	3	4	2	4	7	2	5	3	2	1	3	4	2	3
ERX2465194	2	7	3	6	4	4	4	2	4	4	4	4	2	4	7	2	5	2	2	1	3	4	2	3
ERX2465547	2	5	3	5	3	3	2	3	3	4	3	3	5	3	5	2	5	2	2	1	3	4	2	3
ERX2465621	2	1	2	5	1	3	1	4	3	4	3	3	2	3	10	2	5	2	2	1	3	4	2	3
ERX2465229	2	4	3	6	4	3	4	2	4	2	3	4	2	6	6	2	5	3	2	1	3	4	2	3
ERX2465216	2	5	5	4	2	3	4	4	2	1	2	3	2	3	7	1	6	2	2	1	3	4	2	3
ERX2465648	3	5	4	3	3	4	2	4	4	2	4	2	3	1	11	1	5	2	2	1	3	4	2	3
ERX2465412	2	7	4	5	5	4	4	2	-2	4	2	6	-2	4	10	2	5	3	2	1	3	4	2	3
ERX2465678	2	3	2	3	3	2	2	4	4	2	4	2	3	3	7	2	5	2	2	1	3	3	2	3
ERX2465258	2	7	3	3	3	5	4	4	4	4	3	2	6	5	8	2	6	3	2	1	3	4	2	3
ERX2465471	2	5	2	5	3	3	2	3	3	4	3	2	5	2	6	2	3	2	1	1	3	2	2	3
ERX2465160	2	5	1	3	3	3	2	4	3	2	5	2	4	2	4	2	6	2	2	1	3	4	2	2
ERX2465204	5	2	3	4	3	5	2	4	9	2	9	1	10	8	6	2	6	3	1	2	3	3	4	3
ERX2465555	2	4	4	3	1	2	2	5	3	2	5	2	4	2	5	2	5	2	2	1	3	4	2	3
ERX2465448	0	4	3	3	1	3	2	4	3	2	5	2	3	2	4	2	5	2	2	1	3	4	2	3
ERX2465338	3	4	2	2	3	3	2	3	5	4	2	1	2	2	5	2	4	2	2	2	3	3	4	3
ERX2465184	2	5	4	5	3	3	2	3	3	4	4,5	3	5	3	7	2	5	2	1	1	3	4	2	3
ERX2465309	2	6	3	4	3	3	2	3	3	4	3	3	3	3	5	2	3	2	2	1	3	2	2	3
ERX2465244	2	5	1	5	3	3	2	3	3	4	3	3	4	3	7	2	5	2	2	1	3	4	2	1
ERX2465349	2	5	3	6	4	5	4	2	4	2	3	3	2	2	7	2	5	3	2	1	3	4	2	3
ERX2465334	2	5	3	5	3	3	2	3	3	4	3	3	5	3	5	2	5	2	2	1	3	4	1	3
ERX2465339	3	5	3	5	3	2	2	3	3	4	3	3	5	3	6	2	5	2	2	1	3	4	2	3
ERX2465177	2	7	3	3	2	5	3	4	3	4	3	4	7	4	8	2	5	3	2	1	3	4	2	3
ERX2465327	3	5	2	2	3	3	2	3	6	4	2	1	2	3	5	2	4	2	2	2	3	3	4	3
ERX2465395	2	5	3	6	4	5	3	2	4	2	3	4	2	4	7	2	5	3	2	1	3	4	2	3
ERX2465477	2	6	4	4	2	2	3	2	2	1	2	2	2	4	6	1	5	2	2	1	3	4	2	3
ERX2465389	2	1	4	7	4	4	4	2	4	2	2	4	2	3	5	2	5	3	2	1	3	4	2	3
ERX2465573	2	4	3	5	4	5	2	2	4	2	3	4	2	4	8	2	5	3	2	1	3	4	2	3
ERX2465168	2	6	4	3	3	4	2	4	3	2	3	2	3	2	2	2	5	2	2	1	3	4	2	3
ERX2465168	2	6	4	3	3	4	2	4	2	2	3	2	3	2	2	2	5	2	2	1	3	4	2	3
ERX2465549	3	2	4	4	3	2	5	4	3	2	3	2	5	1	9	2	5	2	2	1	3	3	2	3
ERX2465476	2	5	3	6	4	6	1	2	4	2	3	4	2	4	7	2	5	3	2	1	3	4	2	3
ERX2465210	3	5	4	3	3	3	3	4	4	2	3	2	2	1	10	2	6	2	2	1	3	4	2	3
ERX2465396	2	5	3	5	3	3	2	3	3	4	3	3	6	3	7	2	3	2	1	1	3	2	2	3
ERX2465474	2	5	3	6	4	5	3	2	4	2	3	4	2	4	7	2	5	2	2	1	3	4	2	3
ERX2465440	2	7	3	8	2	4	4	5	5	2	1	1	4	3	9	2	5	3	2	1	3	4	3	3
ERX2465296	2	5	3	6	4	5	3	2	4	2	3	4	2	4	7	2	5	3	2	1	3	4	2	3
ERX2465576	2	7	3	3	3	5	4	4	4	4	3	4	6	4	7	2	5	3	2	1	3	4	2	3
ERX2465523	5	2	2	4	2	5	2	4	7	2	2	1	3	5	7	2	6	2	2	2	3	3	5	3
ERX2465171	2	5	2	3	1	3	2	4	2	2	6	2	3	2	5	2	5	2	2	1	3	4	2	3
ERX2465211	2	1	2	5	1	3	1	4	3	4	3	3	2	3	9	2	5	2	1	1	3	4	2	3
ERX2465557	3	5	2	3	4	2	3	4	4	2	3	2	4	1	2	3	5	2	2	1	3	4	2	3
ERX2465254	2	5	2	5	3	3	2	3	3	4	3	2	5	2	6	2	3	2	1	1	3	2	2	3
ERX2465413	2	5	4	5	4	6	1	2	-2	2	3	4	-2	4	7	2	5	3	2	1	3	4	2	3

ERX2465594	2	4	6	3	3	2	2	3	4	2	3	2	3	2	5	2	5	2	1	1	3	4	2	3
ERX2465362	2	1	3	7	1	2	3	5	3	4	3	3	2	2	6	3	5	2	2	1	3	4	2	3
ERX2465289	2	7	3	3	3	5	2	4	4	4	3	2	6	5	8	2	5	4	2	1	4	4	2	3
ERX2465282	2	3	1	3	3	3	2	4	3	2	4	2	2	2	5	2	6	2	2	1	3	4	2	2
ERX2465176	2	7	3	6	4	4	4	2	4	4	4	4	2	4	7	2	5	2	2	1	3	4	2	3
ERX2465288	2	5	1	3	1	3	4	4	2	1	2	2	2	3	7	2	8	2	2	1	2	4	1	5
ERX2465400	2	7	3	6	4	5	5	2	4	2	3	4	2	4	8	2	5	3	2	1	3	4	2	3
ERX2465498	2	5	4	4	3	2	3	3	2	1	2	2	2	3	7	1	7	2	2	1	3	4	2	3
ERX2465372	2	5	2	2	3	3	1	5	4	2	2	1	2	2	5	2	4	2	2	2	3	3	3	3
ERX2465319	2	5	3	6	4	5	3	2	4	2	2	4	2	4	7	2	5	3	2	1	3	4	2	3
ERX2465203	2	5	1	4	1	2	2	4	2	1	2	2	3	3	7	2	7	2	2	1	2	4	1	5
ERX2465559	2	7	3	2	3	5	2	4	5	4	3	2	6	3	8	2	5	2	2	1	3	4	2	3
ERX2465601	2	5	3	3	3	4	4	4	4	4	3	2	6	5	8	2	5	3	2	1	3	4	2	3
ERX2465408	3	6	3	6	2	5	0	3	6	4	1	2	4	3	2	2	4	2	2	2	3	2	3	2
ERX2465380	3	4	3	7	4	3	2	5	-2	4	5	3	-2	3	2	2	4	2	2	1	3	3	2	3
ERX2465418	2	5	2	6	4	5	3	2	4	2	3	4	2	4	8	2	5	3	2	1	3	4	2	3
ERX2465628	2	7	3	3	2	6	4	4	4	2	3	4	6	6	12	2	5	3	2	1	3	4	2	3
ERX2465392	2	1	2	5	1	3	1	4	3	4	3	3	1	3	7	2	5	2	2	1	3	4	2	3
ERX2465548	1	1	1	5	1	3	4	4	3	4	2	3	2	3	7	2	5	2	2	1	3	4	2	3
ERX2465640	2	5	3	3	3	5	4	4	4	4	3	2	6	5	8	2	5	3	2	1	3	4	2	3
ERX2465653	2	6	1	4	2	4	2	4	2	1	2	2	3	3	2	2	6	2	1	1	3	4	2	3
ERX2465465	2	1	4	7	4	4	4	2	4	2	2	4	2	3	5	2	5	3	2	1	3	4	2	3
ERX2465255	3	5	3	4	3	3	3	2	4	2	3	2	2	1	6	2	5	2	2	1	3	4	2	3
ERX2465335	2	1	4	7	4	4	4	2	4	2	2	4	2	3	5	2	5	3	2	1	3	4	2	3
ERX2465209	5	2	3	4	3	6	2	4	6	1	9	1	3	6	6	2	6	3	2	2	3	3	1	3
ERX2465461	2	6	3	3	3	5	4	4	4	4	3	2	5	4	7	2	5	3	2	1	3	4	2	3
ERX2465553	5	2	2	4	3	4	1	4	4	2	2	1	8	10	7	2	6	2	2	2	1	3	6	3
ERX2465688	3	5	3	4	3	3	3	2	4	2	3	2	2	1	6	2	5	2	2	1	3	4	2	3
ERX2465540	2	5	3	5	3	4	2	3	3	4	3	3	5	3	5	2	5	2	2	1	3	4	2	3
ERX2465672	2	6	3	3	3	5	4	4	2	4	3	2	3	4	7	2	5	3	2	1	3	4	2	3
ERX2465324	4	2	3	4	3	5	2	4	10	2	6	1	6	6	4	2	6	3	1	2	1	3	4	3
ERX2465508	2	5	3	6	3	3	2	3	3	4	3	2	6	2	7	2	3	2	1	1	3	2	2	3
ERX2465420	0	5	3	3	3	3	2	4	3	2	3	2	3	2	5	2	6	2	2	1	3	4	2	3
ERX2465533	5	2	2	4	3	4	1	4	4	2	2	1	1	10	8	2	6	3	2	2	3	3	6	3
ERX2465660	2	5	3	6	4	5	3	2	4	2	3	4	2	4	7	2	5	3	2	1	3	4	2	3
ERX2465198	2	5	3	6	4	5	3	2	4	2	3	4	2	4	7	2	5	3	2	1	3	4	2	3
ERX2465603	3	5	2	2	3	3	0	5	4	4	2	1	2	1	5	2	4	2	2	2	2	3	5	3
ERX2465361	2	1	4	7	4	4	3	2	2	2	2	4	2	4	5	2	5	3	2	1	3	4	2	3
ERX2465526	2	3	4	3	2	3	2	4	2	2	3	2	5	2	7	2	5	2	2	1	3	4	2	3
ERX2465346	2	6	4	4	3	4	4	4	2	2	6	2	3	2	5	2	5	2	2	1	2	4	2	3
ERX2465262	2	5	6	4	3	2	3	4	2	1	2	2	2	3	7	1	6	2	2	1	3	4	2	3
ERX2465330	2	4	3	5	4	5	2	2	4	2	3	4	2	4	8	2	5	3	2	1	3	4	2	3
ERX2465192	5	2	3	4	3	5	2	3	6	1	4	1	2	15	5	2	10	2	2	2	3	3	4	3
ERX2465427	5	2	2	4	3	4	1	4	4	2	2	1	1	10	8	2	6	3	2	2	3	3	6	3

ERX2465222	2	5	4	3	3	3	2	3	3	2	1	1	5	3	7	2	5	2	2	1	2	4	2	3
ERX2465302	2	5	1	4	1	2	4	4	2	1	2	2	3	3	8	2	6	2	2	1	2	4	1	5
ERX2465236	2	7	3	3	3	5	2	4	4	4	3	2	4	5	4	2	5	3	2	1	3	4	2	3
ERX2465684	2	7	3	2	3	4	4	4	4	4	3	2	6	5	8	2	5	4	2	1	3	4	2	3
ERX2465253	2	1	2	5	1	3	3	4	2	4	5	5	1	3	8	2	4	2	2	1	3	4	3	1
ERX2465633	2	5	3	6	4	5	3	2	4	2	3	4	2	4	7	2	5	3	2	1	3	4	2	3
ERX2465206	2	1	4	7	4	4	4	2	4	2	2	4	2	3	5	2	5	3	2	1	3	4	2	3
ERX2465227	2	7	2	4	3	4	1	4	2	2	5	2	3	2	5	2	4	2	2	1	3	4	2	3
ERX2465454	3	2	2	4	3	4	1	4	4	2	2	1	8	6	7	2	6	3	2	2	3	3	6	3
ERX2465223	2	5	4	3	1	4	2	4	3	2	3	2	3	2	2	2	5	2	2	1	3	4	2	3
ERX2465486	2	4	1	3	1	3	4	4	2	1	2	2	4	3	7	2	6	2	2	1	2	4	1	5
ERX2465270	2	5	3	6	4	5	3	2	4	2	3	4	2	4	7	2	5	3	2	1	3	4	2	3
ERX2465185	2	5	4	3	1	3	2	4	2	2	5	0	3	2	4	2	5	2	2	1	3	4	2	3
ERX2465422	2	5	3	5	3	3	2	3	3	4	3	3	5	3	5	2	5	2	2	1	3	4	2	3
ERX2465495	3	5	2	2	3	3	2	5	6	4	2	1	2	3	5	2	4	2	2	2	4	3	5	3
ERX2465188	2	5	3	5	3	2	2	3	3	4	3	2	6	2	7	2	3	2	1	1	3	2	1	3
ERX2465278	2	5	2	6	4	5	3	2	4	2	3	4	2	4	7	2	5	3	2	1	3	4	2	3
ERX2465662	2	4	3	5	3	3	2	3	3	4	3	3	4	3	4	2	5	2	2	1	3	4	2	3
ERX2465337	2	4	1	4	2	3	4	4	2	1	2	2	4	3	8	2	6	2	2	1	3	4	1	5
ERX2465453	2	5	3	4	3	3	4	4	2	2	1	2	4	4	7	2	6	2	2	1	3	4	2	1
ERX2465647	5	2	2	4	3	4	1	4	4	2	2	1	8	11	4	2	6	3	2	2	3	3	6	3
ERX2465186	2	5	3	5	3	3	2	3	3	4	3	3	6	3	7	2	3	2	2	1	3	2	2	3
ERX2465307	2	5	3	2	3	3	2	4	3	2	3	2	2	3	7	2	5	2	2	1	3	4	1	2
ERX2465162	2	5	4	4	3	2	3	3	2	1	2	2	2	3	7	1	7	2	2	1	3	4	2	3
ERX2465577	2	7	3	3	3	5	4	4	4	4	3	2	6	7	6	2	5	3	2	1	3	4	2	3
ERX2465504	2	5	5	3	1	3	2	4	3	2	5	2	3	2	5	2	5	2	2	1	3	4	2	3
ERX2465623	2	4	4	2	1	3	2	2	3	2	5	2	4	2	5	2	5	2	2	1	3	4	2	3
ERX2465394	2	4	2	3	4	3	2	4	2	2	3	2	4	2	4,5	2	5	2	2	1	3	4	2	2
ERX2465360	2	5	2	4	3	3	1	4	4	2	4	2	3	3	3	2	3	2	2	1	3	4	2	2
ERX2465641	2	5	5	4	3	2	3	2	2	1	2	2	2	3	6	1	5	2	2	1	3	4	2	2
ERX2465597	2	4	1	4	1	3	4	4	2	1	2	2	5	3	6	2	6	2	2	1	2	2	1	5
ERX2465516	5	1	2	4	2	5	2	4	7	2	2	1	7	8	7	2	6	2	2	2	3	3	5	3
ERX2465438	2	3	2	3	4	3	2	4	1	2	3	2	3	2	5	2	5	2	2	1	3	4	2	3
ERX2465689	2	7	3	6	3	4	4	2	4	4	4	4	2	4	7	2	5	2	2	1	3	4	2	3
ERX2465652	2	7	3	6	4	5	4	2	-2	2	3	3	-2	4	8	2	5	3	2	1	3	4	2	3
ERX2465466	5	2	3	4	3	6	2	4	6	1	4	1	3	6	6	2	6	2	2	2	3	3	4	3
ERX2465646	2	5	3	6	4	5	3	2	4	2	3	4	2	4	7	2	5	3	2	1	2	4	2	3
ERX2465215	2	8	4	4	3	4	5	4	2	2	4	2	3	2	5	2	5	2	2	1	2	4	2	3
ERX2465215	2	8	5	4	3	4	5	4	2	2	4	2	3	2	5	2	5	2	2	1	2	4	2	3
ERX2465234	2	5	3	5	3	3	2	3	3	4	2	3	2	3	7	2	5	2	2	1	3	4	2	3
ERX2465534	2	5	3	5	3	3	2	3	3	4	3	3	3	2	7	2	5	2	2	1	4	4	2	3
ERX2465614	2	1	2	5	1	3	1	4	3	4	3	3	1	3	7	2	5	2	1	1	3	4	2	3
ERX2465583	2	6	3	2	3	3	2	4	3	2	3	2	2	5	7	2	5	2	2	1	3	2	1	2
ERX2465200	2	7	2	6	4	4	4	2	3	2	3	2	2	4	8	2	5	3	2	1	3	4	2	3
ERX2465391	2	5	3	5	4	5	3	2	-2	2	3	4	2	3	8	2	5	3	2	1	3	4	2	3

ERX2465449	2	5	2	6	3	5	3	2	-2	2	3	4	5	6	6	2	7	3	2	1	3	4	2	3
ERX2465588	2	5	2	3	3	3	1	4	3	2	4	2	3	3	4	2	3	2	2	1	3	4	2	2
ERX2465242	2	5	2	2	3	3	2	4	3	2	3	2	3	3	7	2	5	2	2	1	3	4	1	3
ERX2465231	5	2	2	1	3	4	1	4	4	2	2	1	9	9	7	2	6	3	2	2	3	3	6	3
ERX2465568	2	1	4	7	4	4	4	2	4	2	2	4	2	3	5	2	5	3	2	1	3	4	2	3
ERX2465625	2	1	4	7	4	4	4	2	4	2	2	4	2	3	5	2	5	3	2	1	3	4	2	3
ERX2465315	2	7	3	4	3	4	4	2	3	2	3	2	2	4	7	2	5	3	2	1	3	4	2	2
ERX2465543	2	5	3	3	3	3	2	4	3	2	5	2	3	2	5	2	6	2	2	1	3	4	2	3
ERX2465615	2	6	3	6	3	3	2	3	3	4	3	3	4	2	7	2	5	2	2	1	3	4	2	3
ERX2465286	2	8	3	3	3	5	4	4	3	4	4	2	5	3	8	2	5	3	2	1	3	4	2	3
ERX2465485	2	5	1	2	3	2	4	4	3	2	3	2	4	2	7	2	5	2	2	1	3	4	2	3
ERX2465492	2	3	4	4	4	2	3	6	2	1	2	2	2	3	5	1	6	2	2	1	3	4	2	3
ERX2465221	2	5	3	3	3	5	4	4	4	4	3	2	6	5	8	2	5	2	2	1	3	4	2	3
ERX2465624	4	2	1	4	3	5	2	4	7	2	4	1	8	6	6	2	6	3	2	3	4	3	4	3
ERX2465343	2	5	4	5	3	3	2	3	3	4	4	3	6	5	7	2	5	2	2	1	3	4	2	3
ERX2465190	2	2	3	3	4	3	2	4	2	2	3	2	3	2	3	2	5	2	2	1	3	4	2	2
ERX2465169	2	6	3	7	3	4	2	3	3	4	5	3	2	6	7	2	5	2	2	1	3	4	2	2
ERX2465331	2	5	4	4	2	3	4	2	2	2	1	2	3	2	6	2	6	1	2	1	3	4	2	1
ERX2465268	2	7	3	2	3	5	4	4	4	4	4	2	6	5	6	2	5	4	2	1	3	4	2	3
ERX2465631	2	6	2	7	3	4	2	3	3	4	7	3	2	6	7	2	5	2	2	1	3	4	2	2
ERX2465522	2	5	2	2	3	3	1	4	2	2	3	2	3	2	5	2	6	2	2	1	3	4	2	3
ERX2465357	2	5	2	3	3	4	1	3	3	2	5	2	3	3	4	2	5	2	2	1	3	4	2	2
ERX2465567	2	4	5	3	3	5	2	4	4	4	3	4	4	3	8	2	5	3	2	1	4	4	2	3
ERX2465276	2	7	3	3	3	5	4	4	3	4	3	2	5	5	8	2	5	3	2	1	3	4	2	3
ERX2465473	2	1	4	7	4	4	2	2	4	2	2	4	2	3	5	2	5	3	2	1	3	4	2	3
ERX2465539	2	1	3	5	1	3	3	4	3	4	3	3	2	4	8	2	5	2	2	0	3	4	2	3

Supplementary Table S2. Sequence quality of the 535 sequences according to Breseq software. This supplementary material is hosted by *Eurosurveillance* as supporting information alongside the article [Towards standardisation: comparison of five whole genome sequencing (WGS) analysis pipelines for detection of epidemiologically linked tuberculosis cases], on behalf of the authors, who remain responsible for the accuracy and appropriateness of the content. The same standards for ethics, copyright, attributions and permissions as for the article apply. Supplements are not edited by *Eurosurveillance* and the journal is not responsible for the maintenance of any links or email addresses provided therein.

strain	reads	bases	passed filters	average	longest	mapped	cov	var	%mapped reads
ERX2465512	1484112	180711073	100.0%	121,8	126	0,968	80,9	4,4	98.8%
ERX2465208	1713998	213178275	100.0%	124,4	126	0,973	95,9	5,8	98.8%
ERX2465608	1669847	204288928	100.0%	122,3	126	0,954	100,6	34,1	98.0%
ERX2465437	2607223	303628567	100.0%	116,5	126	0,975	136,1	8,9	98.6%
ERX2465317	2696550	319408541	100.0%	118,5	126	0,944	139,7	8,1	98.5%
ERX2465584	2928791	342924858	100.0%	117,1	126	0,976	155,2	7,8	98.9%
ERX2465264	1499296	187132061	100.0%	124,8	126	0,97	83,9	5	98.7%
ERX2465292	1427504	172675841	100.0%	121	126	0,971	76,8	4,8	98.6%
ERX2465384	2937000	350313164	100.0%	119,3	126	0,97	157,2	6,4	98.7%
ERX2465263	3912809	474043691	100.0%	121,2	126	0,973	216,1	10,5	98.8%
ERX2465424	4071543	467586791	100.0%	114,8	126	0,967	210,4	10,8	98.6%

ERX2465507	3386647	384504872	100.0%	113,5	126	0,959	168,4	11,2	98.5%
ERX2465312	6056757	699201206	100.0%	115,4	126	0,971	319,4	12,4	99.0%
ERX2465675	2213733	267642795	100.0%	120,9	126	0,976	119,9	7,6	98.5%
ERX2465673	2905485	355748172	100.0%	122,4	126	0,977	158,8	8,5	98.6%
ERX2465314	5021362	590596786	100.0%	117,6	126	0,977	267,8	10	98.5%
ERX2465525	2070869	246689990	100.0%	119,1	126	0,967	110,8	6,2	98.9%
ERX2465411	1848078	229948669	100.0%	124,4	126	0,968	102,6	6,2	98.3%
ERX2465180	4618207	531607592	100.0%	115,1	126	0,954	238,4	8,1	98.6%
ERX2465481	2000000	235432087	100.0%	117,7	126	0,98	102,7	6,9	98.9%
ERX2465591	1595548	196556391	100.0%	123,2	126	0,96	96,7	22,4	98.5%
ERX2465293	1999989	248342101	100.0%	124,2	126	0,967	111	6,1	98.3%
ERX2465635	1588769	190712284	100.0%	120	126	0,97	85	5,4	98.5%
ERX2465441	2000000	248760439	100.0%	124,4	126	0,976	107,9	7,6	98.8%
ERX2465587	3550402	412216489	100.0%	116,1	126	0,957	186,3	10,2	98.6%
ERX2465503	3498359	410185605	100.0%	117,3	126	0,974	186,7	8	98.8%
ERX2465399	2780848	320711084	100.0%	115,3	126	0,967	143,4	9,4	98.9%
ERX2465593	1815678	221473765	100.0%	122	126	0,98	97,6	6,6	98.6%
ERX2465373	2373399	282326095	100.0%	119	126	0,947	125,9	5,3	98.7%
ERX2465323	3588955	417791547	100.0%	116,4	126	0,964	186,7	11,7	98.9%
ERX2465571	3528006	404421922	100.0%	114,6	126	0,969	180	11	98.5%
ERX2465239	3142894	359627154	100.0%	114,4	126	0,977	159,6	10,2	98.6%
ERX2465304	2129153	256734935	100.0%	120,6	126	0,979	110,5	6,5	98.1%
ERX2465610*	2000000	232922216	100.0%	116,5	126	0,43	42,9	4,1	98.3%
ERX2465228	2000000	212976647	100.0%	106,5	126	0,981	95	4	98.6%
ERX2465589	2000000	238271822	100.0%	119,1	126	0,98	103,6	6,8	98.3%
ERX2465187	2000000	237653244	100.0%	118,8	126	0,982	102,4	7,9	98.6%
ERX2465283	2000000	237222199	100.0%	118,6	126	0,982	109	7	98.9%
ERX2465664*	2000000	236687693	100.0%	118,3	126	0,651	65,6	6,6	98.3%
ERX2465579	2000000	244579772	100.0%	122,3	126	0,98	107,2	7,1	98.8%
ERX2465405	3275050	385980690	100.0%	117,9	126	0,972	174,9	9,4	98.7%
ERX2465470	2000000	234109273	100.0%	117,1	126	0,979	106,9	6,5	98.8%
ERX2465463	2165974	260973573	100.0%	120,5	126	0,981	118,6	8	98.8%
ERX2465671	3054342	359924352	100.0%	117,8	126	0,816	134,5	8,6	98.7%
ERX2465505	2000000	235950195	100.0%	118	126	0,982	107	6,3	98.8%
ERX2465275	3838047	447861543	100.0%	116,7	126	0,977	203,4	11,1	98.8%
ERX2465563	3089654	366846062	100.0%	118,7	126	0,938	161,8	7,6	98.5%
ERX2465363	5468211	630730824	100.0%	115,3	126	0,962	283,5	10,4	98.9%
ERX2465161	4581326	516726019	100.0%	112,8	126	0,972	236,4	9,3	98.8%
ERX2465596	4017816	472250013	100.0%	117,5	126	0,98	212,4	11,1	98.5%
ERX2465487	2343109	289081002	100.0%	123,4	126	0,965	129,4	7	98.4%
ERX2465514*	2000000	243667290	100.0%	121,8	126	0,099	8,2	2,8	98.7%
ERX2465580	4002101	457213646	100.0%	114,2	126	0,978	208,3	8	98.6%
ERX2465502	2248930	276837747	100.0%	123,1	126	0,972	124,7	5,2	98.3%
ERX2465619	4491031	522188677	100.0%	116,3	126	0,94	228,8	10	98.6%
ERX2465313	2647389	308219044	100.0%	116,4	126	0,976	133,9	9,5	98.9%
ERX2465409	1284216	152892939	100.0%	119,1	126	0,975	68,2	4,3	98.5%
ERX2465219	2902709	344315884	100.0%	118,6	126	0,866	138,9	6,6	98.7%
ERX2465266	1868551	232939348	100.0%	124,7	126	0,972	106,5	6,2	98.5%

ERX2465531	5207378	564449230	100.0%	108,4	126	0,975	252,6	12,1	98.5%
ERX2465397	3749338	440549857	100.0%	117,5	126	0,969	197,4	8	98.5%
ERX2465303	3378025	399599407	100.0%	118,3	126	0,975	183,8	9,6	98.7%
ERX2465245	1350926	167188313	100.0%	123,8	126	0,861	66,2	4,1	98.5%
ERX2465674	1769768	216680466	100.0%	122,4	126	0,978	97,5	6,1	98.7%
ERX2465509	3305502	394431568	100.0%	119,3	126	0,981	179,7	9,9	98.6%
ERX2465235	4653712	564097049	100.0%	121,2	126	0,98	249,9	13,9	99.1%
ERX2465300	1984696	236046084	100.0%	118,9	126	0,975	104,7	8,4	98.4%
ERX2465199	3042584	362900739	100.0%	119,3	126	0,975	164,7	8,9	98.8%
ERX2465551	2703442	319299991	100.0%	118,1	126	0,929	138,3	8,2	98.8%
ERX2465170	4016888	477199740	100.0%	118,8	126	0,955	212,1	8,6	98.8%
ERX2465622	1782418	215171589	100.0%	120,7	126	0,976	96,4	5,7	98.3%
ERX2465686	5096170	582089147	100.0%	114,2	126	0,981	269	11,4	98.7%
ERX2465260	1999998	226014260	100.0%	113	126	0,98	104,1	6,1	98.9%
ERX2465390	3870328	424091220	100.0%	109,6	126	0,841	171,9	6,9	98.6%
ERX2465458	1393416	171636644	100.0%	123,2	126	0,975	77,1	5	98.7%
ERX2465213	2391517	285867016	100.0%	119,5	126	0,977	128,8	8,1	98.6%
ERX2465529	1825703	225299866	100.0%	123,4	126	0,969	100,5	6,3	98.7%
ERX2465371	2176983	270070470	100.0%	124,1	126	0,978	122,3	7,1	98.4%
ERX2465506	2000000	234501749	100.0%	117,3	126	0,982	99,6	7,8	98.6%
ERX2465659	1517443	180823747	100.0%	119,2	126	0,98	81,7	5,4	98.7%
ERX2465520	1951586	226080829	100.0%	115,8	126	0,977	98,2	5,4	98.6%
ERX2465537	1780701	212974701	100.0%	119,6	126	0,972	95,5	7,4	98.5%
ERX2465259	1744205	208864846	100.0%	119,7	126	0,974	92,3	5,7	98.6%
ERX2465336	1493164	178225594	100.0%	119,4	126	0,976	80,1	4,7	98.6%
ERX2465457	1918441	229857541	100.0%	119,8	126	0,975	104,9	6	98.8%
ERX2465556	2128641	265310423	100.0%	124,6	126	0,972	124,8	14	98.6%
ERX2465365	1712894	209210762	100.0%	122,1	126	0,972	96,4	9,1	98.8%
ERX2465256	1862575	224183295	100.0%	120,4	126	0,964	87,9	9	98.9%
ERX2465683	2809697	351246450	100.0%	125	126	0,98	160,4	6,3	98.6%
ERX2465374	1832424	215700797	100.0%	117,7	126	0,977	70,6	7	98.7%
ERX2465345	1927978	231309866	100.0%	120	126	0,964	105,9	13,2	98.8%
ERX2465575	1851365	222193699	100.0%	120	126	0,974	99,3	5,1	98.4%
ERX2465247	1817944	214226145	100.0%	117,8	126	0,972	95,3	5,8	98.7%
ERX2465632	1938436	231828279	100.0%	119,6	126	0,974	102,9	7,1	98.4%
ERX2465536	1838047	219294932	100.0%	119,3	126	0,967	102,1	9,3	99.0%
ERX2465585	2012461	241514957	100.0%	120	126	0,973	76,3	7,1	98.5%
ERX2465428	1958599	233279103	100.0%	119,1	126	0,973	98,9	7,3	98.6%
ERX2465455	1945258	232505191	100.0%	119,5	126	0,976	103,5	5,7	99.0%
ERX2465205	1605917	190816528	100.0%	118,8	126	0,977	83,7	5,3	98.4%
ERX2465600	1287088	155753537	100.0%	121	126	0,969	73,5	10,4	98.8%
ERX2465444	1591101	191867919	100.0%	120,6	126	0,972	85,1	26	98.6%
ERX2465340	1974797	240388220	100.0%	121,7	126	0,978	111,4	9,6	98.6%
ERX2465582	1787718	219251459	100.0%	122,6	126	0,973	98,3	5,5	98.7%
ERX2465401	968340	117736882	100.0%	121,6	126	0,976	52,7	3,5	98.6%
ERX2465450	4081953	490354191	100.0%	120,1	126	0,981	223,9	13,5	98.7%
ERX2465443	4566862	561935977	100.0%	123	126	0,971	252,3	12,9	98.6%
ERX2465528	890410	108980270	100.0%	122,4	126	0,969	48,8	7,7	98.8%

ERX2465431	1250056	151849903	100.0%	121,5	126	0,967	67,8	5	98.8%
ERX2465367	2707302	327193484	100.0%	120,9	126	0,975	146,2	8,8	98.6%
ERX2465251	2701739	329923973	100.0%	122,1	126	0,98	148	8,2	98.4%
ERX2465479	1461464	175813194	100.0%	120,3	126	0,959	78,1	6,4	98.8%
ERX2465513	853889	104254034	100.0%	122,1	126	0,911	42	7,5	98.8%
ERX2465377	2588917	321150690	100.0%	124	126	0,977	143,8	7,8	98.7%
ERX2465687	1364020	170843778	100.0%	125,3	126	0,973	78	5,6	98.4%
ERX2465642	1836039	219853832	100.0%	119,7	126	0,972	69,2	19,3	98.2%
ERX2465273	2149348	266558726	100.0%	124	126	0,952	117,2	6,7	98.5%
ERX2465578	961753	119306677	100.0%	124,1	126	0,919	50,7	3,4	98.7%
ERX2465524	801248	97315677	100.0%	121,5	126	0,973	44,5	5,2	98.8%
ERX2465375	1840917	223667447	100.0%	121,5	126	0,98	101,2	5,9	98.8%
ERX2465201*	1900385	225225056	100.0%	118,5	126	0,652	68,8	4	98.5%
ERX2465562	3072588	365572803	100.0%	119	126	0,98	165,1	8,2	98.6%
ERX2465301	2504057	304242974	100.0%	121,5	126	0,963	143,9	19,9	98.9%
ERX2465685	2268886	272967515	100.0%	120,3	126	0,976	126,6	10,1	98.7%
ERX2465386	1829715	220458061	100.0%	120,5	126	0,972	132,4	56,6	98.7%
ERX2465196	1266379	150841511	100.0%	119,1	126	0,976	68,7	6,6	98.8%
ERX2465226	1774859	212824371	100.0%	119,9	126	0,978	97,9	7	98.6%
ERX2465480	3742055	454952257	100.0%	121,6	126	0,978	204,7	8,3	98.7%
ERX2465388	2000000	230450657	100.0%	115,2	126	0,981	105	8,1	99.1%
ERX2465643	2571554	308378437	100.0%	119,9	126	0,972	138,3	36,5	98.6%
ERX2465554	1978555	236012235	100.0%	119,3	126	0,977	106,2	4,8	98.9%
ERX2465250	1327400	162739997	100.0%	122,6	126	0,969	71,6	5,9	98.7%
ERX2465280	2403531	287501447	100.0%	119,6	126	0,979	128,2	6,3	98.7%
ERX2465620	2159755	256446003	100.0%	118,7	126	0,951	113,1	6	99.0%
ERX2465570	2000000	235088983	100.0%	117,5	126	0,982	102,9	6,9	98.7%
ERX2465311	2000000	238155906	100.0%	119,1	126	0,96	105	6,1	98.6%
ERX2465321	2000000	236432474	100.0%	118,2	126	0,834	91,6	5,3	98.4%
ERX2465510	2000000	229976408	100.0%	115	126	0,983	104	7,1	98.6%
ERX2465238	3024337	361531559	100.0%	119,5	126	0,965	103,1	28,8	98.4%
ERX2465329	2977389	358308620	100.0%	120,3	126	0,967	119,5	29,5	98.3%
ERX2465415	2935141	350727952	100.0%	119,5	126	0,969	96,6	28,8	98.3%
ERX2465189	2821170	336638805	100.0%	119,3	126	0,971	135,2	28,5	98.5%
ERX2465616	2125124	255726057	100.0%	120,3	126	0,965	116,7	16	98.6%
ERX2465590	2730283	329352896	100.0%	120,6	126	0,966	133,9	38	98.3%
ERX2465414	2758615	330310513	100.0%	119,7	126	0,959	88,9	23,7	98.2%
ERX2465518	2035834	244918297	100.0%	120,3	126	0,945	52,1	12	98.4%
ERX2465446	1755795	210590578	100.0%	119,9	126	0,926	94,9	10,5	98.9%
ERX2465416	2886775	347565794	100.0%	120,4	126	0,966	101,1	31,1	98.3%
ERX2465439	2469251	295677727	100.0%	119,7	126	0,971	142,8	35,6	98.5%
ERX2465159	2510136	301812643	100.0%	120,2	126	0,966	102,1	24,6	98.3%
ERX2465654	2080393	249502172	100.0%	119,9	126	0,965	70,2	13,9	98.2%
ERX2465484	2233926	269470837	100.0%	120,6	126	0,968	125,9	18	98.6%
ERX2465344	1840006	220954944	100.0%	120,1	126	0,946	100,8	12,4	98.7%
ERX2465666	2000000	228145208	100.0%	114,1	126	0,981	99,7	7,8	98.5%
ERX2465364	3551619	414496950	100.0%	116,7	126	0,978	185,6	8,9	98.3%
ERX2465281	2000000	230015127	100.0%	115	126	0,982	104,2	6,9	98.7%

ERX2465566	2507514	300326197	100.0%	119,8	126	0,971	154,6	39	98.5%
ERX2465612	2000000	229080272	100.0%	114,5	126	0,98	92,2	8,1	98.4%
ERX2465667*	1999999	223567205	100.0%	111,8	126	0,085	1,3	1,6	99.6%
ERX2465681	2000000	234731081	100.0%	117,4	126	0,926	95,1	6,5	98.5%
ERX2465285	2000000	233920341	100.0%	117	126	0,979	104,4	6,8	99.0%
ERX2465350	1999999	232316995	100.0%	116,2	126	0,98	99,7	6,9	98.6%
ERX2465241	1999999	233601095	100.0%	116,8	126	0,982	98,1	7,3	98.3%
ERX2465592	3044578	364560449	100.0%	119,7	126	0,969	171,6	18,1	99.0%
ERX2465611	986011	116626525	100.0%	118,3	126	0,952	49,5	10,2	98.9%
ERX2465532	839676	99321229	100.0%	118,3	126	0,946	41,4	7,9	98.7%
ERX2465166	1425380	167165637	100.0%	117,3	126	0,956	53,5	15,9	98.5%
ERX2465404	1245724	147044410	100.0%	118	126	0,952	57	16,4	98.3%
ERX2465433	2317647	290154421	100.0%	125,2	126	0,978	131,3	7,9	98.9%
ERX2465432	1804416	214501060	100.0%	118,9	126	0,943	69,6	13,1	98.4%
ERX2465356	2000000	226631246	100.0%	113,3	126	0,983	102,8	5,6	98.6%
ERX2465214	2000000	224602569	100.0%	112,3	126	0,979	99,7	6	98.6%
ERX2465218	2000000	224816120	100.0%	112,4	126	0,98	101,6	6,2	98.2%
ERX2465451	723311	83670292	100.0%	115,7	126	0,942	37,2	6,2	98.6%
ERX2465284	3051483	381746089	100.0%	125,1	126	0,977	169,6	9,6	99.0%
ERX2465225	2000000	234947899	100.0%	117,5	126	0,983	100	8,6	98.7%
ERX2465419	2000000	234677832	100.0%	117,3	126	0,985	105,4	5,4	98.7%
ERX2465606	1911836	230238867	100.0%	120,4	126	0,956	73,5	15,4	98.5%
ERX2465328	2000000	223741752	100.0%	111,9	126	0,983	102	4,5	98.6%
ERX2465488	2000000	226602381	100.0%	113,3	126	0,978	102,7	6,5	98.5%
ERX2465639	2944407	346714147	100.0%	117,8	126	0,988	157,8	7,5	99.1%
ERX2465183	1363135	165092770	100.0%	121,1	126	0,96	56,2	16	98.4%
ERX2465478	3040528	355157593	100.0%	116,8	126	0,978	161,3	7,6	98.8%
ERX2465464	3551229	403780920	100.0%	113,7	126	0,972	182,9	10,3	99.1%
ERX2465644	2797115	329706443	100.0%	117,9	126	0,977	151,3	6,9	98.7%
ERX2465202	1406238	170979791	100.0%	121,6	126	0,957	72,7	20	98.4%
ERX2465212	1649222	196078887	100.0%	118,9	126	0,972	92,3	8,2	98.9%
ERX2465670	3201008	375367879	100.0%	117,3	126	0,97	196,6	51,6	98.8%
ERX2465406	3144674	376699087	100.0%	119,8	126	0,98	170,1	9,1	98.6%
ERX2465299	1572979	188752265	100.0%	120	126	0,9	21,8	4,9	98.3%
ERX2465501	2405406	283450208	100.0%	117,8	126	0,957	82,2	14,3	98.9%
ERX2465511	3073156	366116273	100.0%	119,1	126	0,985	170	8,8	98.7%
ERX2465157	1907101	229155902	100.0%	120,2	126	0,938	45,4	9,2	98.4%
ERX2465519	1911700	227165799	100.0%	118,8	126	0,931	38,6	8,7	98.1%
ERX2465232	1533091	184008565	100.0%	120	126	0,941	42	12	98.3%
ERX2465663	1698117	205949872	100.0%	121,3	126	0,953	63,1	12,9	98.2%
ERX2465167	2774972	323695664	100.0%	116,6	126	0,977	146,7	7,7	98.7%
ERX2465462	2961024	353640124	100.0%	119,4	126	0,978	157,4	9,5	98.6%
ERX2465627	2589944	303299573	100.0%	117,1	126	0,947	134	7,8	98.7%
ERX2465308*	1557668	181317082	100.0%	116,4	126	0,636	55,1	12,7	98.4%
ERX2465661	4266163	500558033	100.0%	117,3	126	0,987	233,3	7,5	98.8%
ERX2465626	1993433	237292139	100.0%	119	126	0,965	109,8	10,4	99.0%
ERX2465581	2286983	269795522	100.0%	118	126	0,977	120,7	8,8	98.5%
ERX2465655	2353679	286205627	100.0%	121,6	126	0,963	198,5	85,8	98.7%

ERX2465500	1962829	235572341	100.0%	120	126	0,97	111,9	15,7	98.7%
ERX2465237	1860820	221978513	100.0%	119,3	126	0,907	44,6	9,9	98.1%
ERX2465669	1762865	210844499	100.0%	119,6	126	0,958	55,3	12,6	98.4%
ERX2465517	1873465	230656624	100.0%	123,1	126	0,98	79,5	6,8	98.5%
ERX2465605	4427592	509576823	100.0%	115,1	126	0,762	180,2	10,5	98.8%
ERX2465447	3516597	424968855	100.0%	120,8	126	0,979	185,2	12,7	98.2%
ERX2465460	2000000	238312701	100.0%	119,2	126	0,98	104,6	7,6	98.4%
ERX2465267	1661813	197198500	100.0%	118,7	126	0,841	89,6	37,1	98.7%
ERX2465521	2068277	246767651	100.0%	119,3	126	0,976	112	5,7	98.6%
ERX2465650	2157025	262106222	100.0%	121,5	126	0,96	103,7	23	98.5%
ERX2465651	1432383	175594472	100.0%	122,6	126	0,947	51,5	16,4	98.5%
ERX2465378	2563293	298934684	100.0%	116,6	126	0,977	135,3	7,1	98.6%
ERX2465191	1999999	245933232	100.0%	123	126	0,978	109,1	7,4	98.5%
ERX2465630	1559101	187446134	100.0%	120,2	126	0,963	80,7	22,4	98.3%
ERX2465469	2245947	265226681	100.0%	118,1	126	0,976	122	6,8	98.7%
ERX2465574	1795979	216486472	100.0%	120,5	126	0,939	34,6	6,7	98.3%
ERX2465352	2148476	259472939	100.0%	120,8	126	0,976	60,8	15,4	98.3%
ERX2465257	2259284	268732797	100.0%	118,9	126	0,978	121,6	6,4	98.7%
ERX2465569	1705028	204648885	100.0%	120	126	0,974	92,4	5,5	99.0%
ERX2465298	1956032	234064372	100.0%	119,7	126	0,945	63,8	14,4	98.1%
ERX2465545	2487693	291785823	100.0%	117,3	126	0,971	116,2	7,9	98.6%
ERX2465679	1530919	182977176	100.0%	119,5	126	0,942	60,5	17,5	98.8%
ERX2465682	1458863	173440613	100.0%	118,9	126	0,972	77,7	5,5	98.6%
ERX2465515	4142652	483500421	100.0%	116,7	126	0,985	219,2	9,7	98.6%
ERX2465657*	2000000	224461519	100.0%	112,2	126	0,597	57	6,6	98.7%
ERX2465272	2041254	244026465	100.0%	119,5	126	0,976	110	5,3	98.5%
ERX2465602	1918265	230404823	100.0%	120,1	126	0,959	96,6	15,1	98.4%
ERX2465535	1904695	228629457	100.0%	120	126	0,966	103,3	13,8	98.7%
ERX2465452	2032953	239369164	100.0%	117,7	126	0,973	98,8	7,8	98.3%
ERX2465617	3326376	381516319	100.0%	114,7	126	0,976	166,6	12,5	98.3%
ERX2465690	2186020	270155681	100.0%	123,6	126	0,97	120,9	6,8	98.7%
ERX2465382	3552117	421119206	100.0%	118,6	126	0,976	190,4	10,4	98.6%
ERX2465351	3727762	439876209	100.0%	118	126	0,971	197,8	12,6	98.5%
ERX2465274	3676254	428123362	100.0%	116,5	126	0,975	156,2	12,1	98.6%
ERX2465316	1999999	234994508	100.0%	117,5	126	0,963	101,3	6,6	98.4%
ERX2465217	3640381	424423617	100.0%	116,6	126	0,974	189,5	10,6	98.5%
ERX2465249	3315897	407746411	100.0%	123	126	0,978	185	9,3	98.7%
ERX2465290	1485495	183341250	100.0%	123,4	126	0,977	83,2	4,3	98.7%
ERX2465164	6005593	718401158	100.0%	119,6	126	0,972	202,9	60	98.2%
ERX2465403	2627092	310726411	100.0%	118,3	126	0,973	140,1	6,8	99.0%
ERX2465348	2000000	248273349	100.0%	124,1	126	0,978	110	6,5	98.4%
ERX2465248	3494061	399381547	100.0%	114,3	126	0,958	176,8	10,6	98.7%
ERX2465224	4621858	542053353	100.0%	117,3	126	0,975	232,8	11,9	98.9%
ERX2465240	1649096	201455239	100.0%	122,2	126	0,86	49,3	9,6	97.9%
ERX2465542	3035398	376474049	100.0%	124	126	0,972	215,4	73,3	98.6%
ERX2465402	3015635	363155207	100.0%	120,4	126	0,958	68,9	15,2	98.2%
ERX2465193	2550655	311980047	100.0%	122,3	126	0,959	61,5	12,5	98.5%
ERX2465370	2747532	340542787	100.0%	123,9	126	0,959	154,5	10,7	98.9%

ERX2465467	3083883	348433716	100.0%	113	126	0,969	136,2	13	98.4%
ERX2465558	1994952	243908528	100.0%	122,3	126	0,973	109,1	6,3	98.5%
ERX2465656	3188840	387964758	100.0%	121,7	126	0,975	175	14,6	98.8%
ERX2465381	2162446	271019384	100.0%	125,3	126	0,976	118,9	6,3	98.2%
ERX2465561	2069702	252146878	100.0%	121,8	126	0,967	115,9	17,5	98.9%
ERX2465295	2484941	300826116	100.0%	121,1	126	0,975	132,9	9,1	98.5%
ERX2465459	2349459	284381722	100.0%	121	126	0,973	128,1	6,7	98.6%
ERX2465342	2383696	289056894	100.0%	121,3	126	0,974	117,5	8,7	98.4%
ERX2465613	1999999	234875726	100.0%	117,4	126	0,973	103,6	7,4	98.7%
ERX2465243	2034350	252469402	100.0%	124,1	126	0,97	116,5	11,3	98.6%
ERX2465421	1762222	218982351	100.0%	124,3	126	0,973	98,2	6	98.5%
ERX2465252	3339675	396493016	100.0%	118,7	126	0,979	175,4	8,5	98.1%
ERX2465265	2036866	252818748	100.0%	124,1	126	0,959	54,4	14,6	98.6%
ERX2465499	2581272	313856436	100.0%	121,6	126	0,963	89,3	26,1	98.3%
ERX2465354	1518817	183127643	100.0%	120,6	126	0,931	22,6	5,3	98.3%
ERX2465423	2819238	336741955	100.0%	119,4	126	0,976	148,9	8,4	98.5%
ERX2465297	2904887	341085571	100.0%	117,4	126	0,975	148,8	8,6	98.4%
ERX2465680	1791264	216698386	100.0%	121	126	0,964	97,7	8,3	98.6%
ERX2465385	2600065	321188173	100.0%	123,5	126	0,974	154,6	37,2	98.6%
ERX2465527	1845404	223915905	100.0%	121,3	126	0,973	106,4	14,2	98.6%
ERX2465182	2574450	319009640	100.0%	123,9	126	0,983	146,2	7,6	98.8%
ERX2465179	3190060	387338492	100.0%	121,4	126	0,973	192,5	46,7	98.8%
ERX2465425	1259416	153398615	100.0%	121,8	126	0,971	67	5	98.7%
ERX2465271	3594608	410432920	100.0%	114,2	126	0,977	176,8	14,1	98.6%
ERX2465493	1662036	203392124	100.0%	122,4	126	0,946	94,8	20,3	98.4%
ERX2465277	2535080	305683587	100.0%	120,6	126	0,948	66,9	16,2	98.2%
ERX2465332	2711792	329833158	100.0%	121,6	126	0,979	145,8	8,3	98.4%
ERX2465426	2663209	325159232	100.0%	122,1	126	0,968	64,9	16,6	98.3%
ERX2465599	3287354	398674787	100.0%	121,3	126	0,972	178,4	32,5	98.4%
ERX2465560	3464266	394951812	100.0%	114	126	0,978	174,5	14,8	98.2%
ERX2465353	3414044	395720369	100.0%	115,9	126	0,977	168,7	13,3	98.4%
ERX2465172	2000000	245043722	100.0%	122,5	126	0,973	111,3	5,5	98.7%
ERX2465483*	2171643	263578057	100.0%	121,4	126	0,597	25,8	9,2	98.5%
ERX2465494	2953724	350988423	100.0%	118,8	126	0,965	156,4	8,4	98.7%
ERX2465325	2000000	234003815	100.0%	117	126	0,971	89,2	7,1	98.4%
ERX2465291	1763714	214056530	100.0%	121,4	126	0,976	95,6	5,5	98.6%
ERX2465322	2618360	324838012	100.0%	124,1	126	0,979	142,7	9	98.5%
ERX2465552	2423718	299904376	100.0%	123,7	126	0,967	135,2	6,3	98.7%
ERX2465178	2224838	269965007	100.0%	121,3	126	0,974	119	8,5	98.4%
ERX2465598	2776842	343493646	100.0%	123,7	126	0,98	155,3	7,7	98.6%
ERX2465435	3327662	396877433	100.0%	119,3	126	0,978	180,3	9,3	98.6%
ERX2465604	3558531	432508811	100.0%	121,5	126	0,98	192,7	11,3	98.3%
ERX2465475	1931511	238966165	100.0%	123,7	126	0,97	109,8	7,3	98.6%
ERX2465658	2413063	290258461	100.0%	120,3	126	0,977	131,7	7,3	98.7%
ERX2465376	2853375	352508986	100.0%	123,5	126	0,978	158,3	7,5	98.5%
ERX2465279	2228216	269610906	100.0%	121	126	0,977	121,6	6,7	98.6%
ERX2465586	2396481	280686712	100.0%	117,1	126	0,977	126,3	7,7	98.4%
ERX2465546	2057002	249141865	100.0%	121,1	126	0,933	106,7	6,4	98.2%

ERX2465676	1647483	200632694	100.0%	121,8	126	0,967	89,7	5,7	98.7%
ERX2465564	2142105	263140277	100.0%	122,8	126	0,971	117,3	7,4	98.6%
ERX2465541	2001241	242077095	100.0%	121	126	0,973	109,5	7,4	98.7%
ERX2465195	2681165	319995629	100.0%	119,3	126	0,971	142,9	8,9	98.6%
ERX2465618	3499334	433403416	100.0%	123,9	126	0,981	198,8	7,7	98.6%
ERX2465230	3428897	416617487	100.0%	121,5	126	0,981	189,2	9,7	98.7%
ERX2465434	1823876	220911379	100.0%	121,1	126	0,971	99,6	7,4	98.8%
ERX2465634	2812544	339587830	100.0%	120,7	126	0,958	147,2	27,6	99.0%
ERX2465565	3253273	402293140	100.0%	123,7	126	0,974	184,3	8,7	99.1%
ERX2465398	1573836	189966702	100.0%	120,7	126	0,969	85,5	4,8	99.0%
ERX2465417	3233163	391397427	100.0%	121,1	126	0,977	177,2	8,8	99.0%
ERX2465287	2996442	357267306	100.0%	119,2	126	0,974	159,2	9,7	98.6%
ERX2465496	2199879	271003889	100.0%	123,2	126	0,974	122,6	7	99.0%
ERX2465197	2865486	346313786	100.0%	120,9	126	0,977	147,6	10,9	98.4%
ERX2465491	2222816	276643583	100.0%	124,5	126	0,973	122,6	6,8	98.4%
ERX2465269*	4244274	499398228	100.0%	117,7	126	0,372	88,4	6,8	98.6%
ERX2465572	2858945	345152877	100.0%	120,7	126	0,975	150,9	8,8	99.0%
ERX2465629	2000000	237996231	100.0%	119	126	0,982	107,9	6,6	98.5%
ERX2465436	3034508	349551745	100.0%	115,2	126	0,977	160,4	10,7	99.1%
ERX2465233	2050688	251697444	100.0%	122,7	126	0,973	114	7	98.9%
ERX2465175	2420267	294758908	100.0%	121,8	126	0,961	130,8	7,6	98.7%
ERX2465607	2318695	281877123	100.0%	121,6	126	0,969	125,4	6,9	98.2%
ERX2465306	3282323	381808632	100.0%	116,3	126	0,975	165,7	12,1	98.4%
ERX2465320	2139812	263711875	100.0%	123,2	126	0,977	119,4	6,3	98.8%
ERX2465472	3415771	420810099	100.0%	123,2	126	0,98	180,5	12,5	98.4%
ERX2465310	1263631	151216103	100.0%	119,7	126	0,971	67,7	4,8	98.7%
ERX2465668	3440203	412143297	100.0%	119,8	126	0,974	183,6	9,3	98.6%
ERX2465430	2049866	246563989	100.0%	120,3	126	0,966	106,6	7,4	98.7%
ERX2465387	3429906	410162619	100.0%	119,6	126	0,974	183,9	9,9	98.5%
ERX2465156	2938218	362151939	100.0%	123,3	126	0,977	163,9	7,4	98.6%
ERX2465333	2779930	325531847	100.0%	117,1	126	0,975	144,5	8,9	98.4%
ERX2465442	2365931	293416067	100.0%	124	126	0,979	129,5	7	98.8%
ERX2465609	1058644	129671410	100.0%	122,5	126	0,975	58,2	3,8	98.5%
ERX2465468	3208935	383003896	100.0%	119,4	126	0,975	170,8	8,5	98.4%
ERX2465174	1048956	128749447	100.0%	122,7	126	0,965	56,8	4,2	98.6%
ERX2465326	2331220	285925577	100.0%	122,7	126	0,962	127,5	7,2	98.8%
ERX2465544	2160919	260432801	100.0%	120,5	126	0,976	116,9	7,4	98.2%
ERX2465368	2136830	263098228	100.0%	123,1	126	0,977	116,4	7,1	98.6%
ERX2465347	1151816	142730610	100.0%	123,9	126	0,966	65,8	7,7	98.7%
ERX2465220	2410365	299824757	100.0%	124,4	126	0,975	134,5	7,5	98.6%
ERX2465181	2014675	243764215	100.0%	121	126	0,972	108,4	6,2	99.0%
ERX2465158	2365302	291675766	100.0%	123,3	126	0,975	132,4	6,8	98.9%
ERX2465207	2462624	305629467	100.0%	124,1	126	0,946	134	7	98.8%
ERX2465379	1721281	210030452	100.0%	122	126	0,962	85,7	21,8	98.1%
ERX2465358	2985932	365942579	100.0%	122,6	126	0,981	165,5	9	98.8%
ERX2465530	1753930	217061206	100.0%	123,8	126	0,964	97,3	5	98.6%
ERX2465366	2128892	262396552	100.0%	123,3	126	0,979	119,7	4,9	98.8%
ERX2465497	1999999	239029617	100.0%	119,5	126	0,977	105,4	7,4	99.0%

ERX2465429	2335230	288172370	100.0%	123,4	126	0,975	129,7	8,2	99.0%
ERX2465305	2536018	310350048	100.0%	122,4	126	0,978	130,4	8,5	98.0%
ERX2465341	2663394	326949882	100.0%	122,8	126	0,979	151,7	7,8	99.1%
ERX2465163	755568	93630385	100.0%	123,9	126	0,854	37,1	2,5	98.6%
ERX2465538*	1506579	179215384	100.0%	119	126	0,634	53,2	4,2	98.5%
ERX2465649*	1832831	226071639	100.0%	123,3	126	0,501	52,8	4,3	99.0%
ERX2465383	1581557	191487573	100.0%	121,1	126	0,966	85,4	5,5	98.9%
ERX2465482	3676367	434149051	100.0%	118,1	126	0,976	196,5	8,8	99.0%
ERX2465393	1453962	175282015	100.0%	120,6	126	0,953	76,2	5,2	98.6%
ERX2465165	2314734	274773813	100.0%	118,7	126	0,849	109,1	6	98.5%
ERX2465637	2187610	262599664	100.0%	120	126	0,917	111,8	6,9	98.7%
ERX2465261	2501557	302516014	100.0%	120,9	126	0,979	137,7	8,2	98.7%
ERX2465489	2876506	340343800	100.0%	118,3	126	0,967	146,5	11,1	98.4%
ERX2465456*	3997583	468057252	100.0%	117,1	126	0,212	47,1	3,6	98.5%
ERX2465410	2193836	263997406	100.0%	120,3	126	0,981	119	7,7	98.7%
ERX2465407	1723073	207234473	100.0%	120,3	126	0,976	92,8	6,2	98.5%
ERX2465636	2007403	244851433	100.0%	122	126	0,936	111,8	14,9	98.7%
ERX2465595	1521566	189168838	100.0%	124,3	126	0,972	86,3	4	98.8%
ERX2465550	2096533	253130418	100.0%	120,7	126	0,975	114,2	6,7	98.2%
ERX2465638	2692285	330374684	100.0%	122,7	126	0,981	150,9	7	98.8%
ERX2465445	1566575	193622887	100.0%	123,6	126	0,887	79,3	4,5	98.7%
ERX2465665	2193120	260633201	100.0%	118,8	126	0,949	114,3	6,1	98.3%
ERX2465294	1569267	190049228	100.0%	121,1	126	0,969	84,5	5,9	99.0%
ERX2465677	2676090	316191090	100.0%	118,2	126	0,971	139,3	9,3	98.4%
ERX2465369	1879908	226881691	100.0%	120,7	126	0,977	103,1	7,1	98.8%
ERX2465490*	2292059	275378061	100.0%	120,1	126	0,485	63,6	4,1	98.7%
ERX2465173	1691617	205058677	100.0%	121,2	126	0,876	82,9	5,6	98.6%
ERX2465318	2175300	255132131	100.0%	117,3	126	0,978	113,3	8,3	98.6%
ERX2465359*	2000000	233395513	100.0%	116,7	126	0,7	77,8	4,3	99.0%
ERX2465246	1676626	206168184	100.0%	123	126	0,946	89,8	5,7	98.7%
ERX2465355	1547265	187024677	100.0%	120,9	126	0,968	83	5,4	98.5%
ERX2465645*	2840116	330181972	100.0%	116,3	126	0,438	65,6	5,5	98.5%
ERX2465194	1655200	195413129	100.0%	118,1	126	0,908	82	5,8	98.5%
ERX2465547	943511	117218739	100.0%	124,2	126	0,968	52,3	3	98.7%
ERX2465621*	4030952	463486973	100.0%	115	126	0,068	15,6	2,4	99.0%
ERX2465229	2200843	266156590	100.0%	120,9	126	0,977	119,7	8,4	98.6%
ERX2465216	2277936	274218545	100.0%	120,4	126	0,988	125,9	7,7	98.7%
ERX2465648	2095161	248325677	100.0%	118,5	126	0,916	106	6,8	98.8%
ERX2465412	1780280	220472193	100.0%	123,8	126	0,946	96,8	4,9	98.6%
ERX2465678	1765212	218184820	100.0%	123,6	126	0,98	99,6	5,4	98.7%
ERX2465258	2665586	324956349	100.0%	121,9	126	0,974	144,8	9,3	98.3%
ERX2465471	1065493	131002923	100.0%	123	126	0,828	50,3	3,7	98.6%
ERX2465160	1322005	159793850	100.0%	120,9	126	0,979	71,5	4,6	98.5%
ERX2465204	1762996	213525459	100.0%	121,1	126	0,73	73,3	4,9	98.6%
ERX2465555	1858552	231444392	100.0%	124,5	126	0,968	103,6	5,7	98.7%
ERX2465448	3369640	415448499	100.0%	123,3	126	0,972	188,6	7,3	98.8%
ERX2465338	1553957	191806496	100.0%	123,4	126	0,879	80,4	4,3	99.0%
ERX2465184	1514003	188293698	100.0%	124,4	126	0,97	85,4	5,1	98.7%

ERX2465309	2859875	350774837	100.0%	122,7	126	0,975	156,6	10,3	98.5%
ERX2465244	2476611	295002291	100.0%	119,1	126	0,937	129,2	7,6	98.7%
ERX2465349*	3468701	394161573	100.0%	113,6	126	0,565	105,7	11,8	98.6%
ERX2465334	1999983	244716719	100.0%	122,4	126	0,969	109,2	5,9	98.6%
ERX2465339	3574338	429509051	100.0%	120,2	126	0,968	194,4	8,2	98.7%
ERX2465177	1971904	233472341	100.0%	118,4	126	0,973	104,6	6	98.3%
ERX2465327	1713840	208471876	100.0%	121,6	126	0,976	95,4	8,1	99.1%
ERX2465395	3197654	384925961	100.0%	120,4	126	0,974	171	11,9	98.4%
ERX2465477	2316113	276084185	100.0%	119,2	126	0,975	125,4	6,7	98.7%
ERX2465389	3258614	391690864	100.0%	120,2	126	0,882	158,8	9,9	98.5%
ERX2465573	2725345	320604852	100.0%	117,6	126	0,976	141,6	8,9	98.7%
ERX2465168	2757527	341387315	100.0%	123,8	126	0,94	148,4	8,8	98.8%
ERX2465549	2200594	264428131	100.0%	120,2	126	0,91	111,7	7	98.7%
ERX2465476	2333728	279720820	100.0%	119,9	126	0,975	126,4	8	98.5%
ERX2465210	1941453	235379039	100.0%	121,2	126	0,831	91,3	5,6	98.7%
ERX2465396	2266380	275283333	100.0%	121,5	126	0,953	121,8	6,7	98.6%
ERX2465474	2879749	333954215	100.0%	116	126	0,972	149,7	9,4	98.5%
ERX2465440	3936558	451520736	100.0%	114,7	126	0,969	202,2	10,8	98.3%
ERX2465296	1543876	191565858	100.0%	124,1	126	0,96	84,5	5,4	98.5%
ERX2465576	1460648	176125404	100.0%	120,6	126	0,969	77,8	5,7	98.4%
ERX2465523	3510085	415195730	100.0%	118,3	126	0,916	177,4	11,4	98.5%
ERX2465171*	5713242	570561290	100.0%	99,9	126	0,03	9	2,8	98.6%
ERX2465211	1141041	142069112	100.0%	124,5	126	0,965	63,1	4,4	99.0%
ERX2465557*	3080958	341103993	100.0%	110,7	126	0,049	8,3	2	98.7%
ERX2465254	2021675	242197001	100.0%	119,8	126	0,973	108	7,1	98.6%
ERX2465413	2869848	334957399	100.0%	116,7	126	0,978	148,3	11,1	98.5%
ERX2465594	2296833	274066190	100.0%	119,3	126	0,964	122,3	7,4	98.6%
ERX2465362	2396211	285341296	100.0%	119,1	126	0,977	127,5	7,9	98.5%
ERX2465289	2200359	262438432	100.0%	119,3	126	0,975	116,3	7,8	98.3%
ERX2465282	1141006	137038281	100.0%	120,1	126	0,975	61,9	4,6	98.9%
ERX2465176	2086205	249345502	100.0%	119,5	126	0,972	111,4	6,6	98.5%
ERX2465288	1931849	232595433	100.0%	120,4	126	0,969	105,6	6,8	98.7%
ERX2465400	3767636	438873299	100.0%	116,5	126	0,966	195,5	12,1	98.6%
ERX2465498*	2590767	295836021	100.0%	114,2	126	0,091	13,1	2,1	98.5%
ERX2465372	2296890	269348816	100.0%	117,3	126	0,967	122,5	7,1	99.1%
ERX2465319	2229778	265390576	100.0%	119	126	0,97	119	6,7	98.5%
ERX2465203	1758390	216849083	100.0%	123,3	126	0,981	99,5	5,8	98.7%
ERX2465559	2372177	280489624	100.0%	118,2	126	0,973	127	6,4	98.4%
ERX2465601	3903473	447519227	100.0%	114,6	126	0,884	186	8,8	98.4%
ERX2465408	2000000	234189724	100.0%	117,1	126	0,975	92,1	7,1	98.9%
ERX2465380	2676761	311253794	100.0%	116,3	126	0,98	142,8	10,2	98.1%
ERX2465418	2477290	290979814	100.0%	117,5	126	0,974	128,6	8,1	98.6%
ERX2465628	2615966	306532657	100.0%	117,2	126	0,963	137	7,1	98.5%
ERX2465392	935055	112682464	100.0%	120,5	126	0,963	51,2	3,9	99.0%
ERX2465548*	3011400	349883110	100.0%	116,2	126	0,218	36,7	3,1	99.0%
ERX2465640	1579965	187213061	100.0%	118,5	126	0,945	82,1	4,9	98.3%
ERX2465653	1892347	229240518	100.0%	121,1	126	0,974	103	6,1	98.7%
ERX2465465	2000000	240594172	100.0%	120,3	126	0,972	107,1	6,3	98.4%

ERX2465255*	2000000	239585961	100.0%	119,8	126	0,557	61,3	4,2	98.8%
ERX2465335	2000000	239292305	100.0%	119,6	126	0,974	105	6,8	98.4%
ERX2465209	2000000	235831197	100.0%	117,9	126	0,976	106,3	7	99.0%
ERX2465461	1999999	236214372	100.0%	118,1	126	0,977	106,3	6,3	98.2%
ERX2465553	2000000	234794799	100.0%	117,4	126	0,98	105,4	5,9	98.5%
ERX2465688	1999999	237009391	100.0%	118,5	126	0,971	101,4	7,6	98.8%
ERX2465540	2000000	241206292	100.0%	120,6	126	0,982	104,8	7,9	98.5%
ERX2465672*	1999996	227786141	100.0%	113,9	126	0,453	49,4	3,6	98.2%
ERX2465324	2000000	235181141	100.0%	117,6	126	0,803	80,8	5,9	98.5%
ERX2465508	1831909	221750868	100.0%	121	126	0,981	99,8	6,7	98.7%
ERX2465420	1939804	240002935	100.0%	123,7	126	0,978	108	6,6	98.7%
ERX2465533	1697646	204526870	100.0%	120,5	126	0,976	91,6	5,1	98.4%
ERX2465660	1999999	237900360	100.0%	119	126	0,98	91,7	8,8	98.3%
ERX2465198	2530541	302463653	100.0%	119,5	126	0,976	134,2	7,4	98.5%
ERX2465603	2361343	275185274	100.0%	116,5	126	0,98	126,5	7,5	99.0%
ERX2465361	2000000	239597759	100.0%	119,8	126	0,977	92,1	9,5	98.3%
ERX2465526	2000000	236832081	100.0%	118,4	126	0,983	92,9	11,2	98.6%
ERX2465346	1213063	146622035	100.0%	120,9	126	0,978	49,3	6,5	98.5%
ERX2465262	2451280	294507055	100.0%	120,1	126	0,981	132,2	8,3	98.7%
ERX2465330	2000000	237035848	100.0%	118,5	126	0,98	102	7,7	98.6%
ERX2465192*	3745979	452731740	100.0%	120,9	126	0,05	10,6	1,8	99.0%
ERX2465427	3779479	441413807	100.0%	116,8	126	0,962	196,6	8,1	98.4%
ERX2465222	1505578	186528218	100.0%	123,9	126	0,967	82,6	5,5	98.7%
ERX2465302	2000000	240004447	100.0%	120	126	0,984	86,1	12,4	98.6%
ERX2465236	2000000	231812873	100.0%	115,9	126	0,981	99,6	7	98.0%
ERX2465684	2000000	238004873	100.0%	119	126	0,98	107,4	6,5	98.1%
ERX2465253	2000000	239025814	100.0%	119,5	126	0,973	105,5	6,6	99.0%
ERX2465633	2000000	239790144	100.0%	119,9	126	0,979	98,6	8,6	98.3%
ERX2465206	2000000	241410065	100.0%	120,7	126	0,979	103	7,7	98.4%
ERX2465227	2000000	238305173	100.0%	119,2	126	0,978	105,1	6,9	98.6%
ERX2465454	2000000	238458602	100.0%	119,2	126	0,979	106,9	6,3	98.5%
ERX2465223	2000000	238439164	100.0%	119,2	126	0,984	107,7	6,8	98.8%
ERX2465486	1472352	180783812	100.0%	122,8	126	0,983	82,9	5,3	98.8%
ERX2465270	2308675	282915938	100.0%	122,5	126	0,704	91	5,6	98.6%
ERX2465185	2402876	295580208	100.0%	123	126	0,966	131,8	7,1	98.7%
ERX2465422	883616	106062568	100.0%	120	126	0,923	44,8	3,6	98.6%
ERX2465495	1899876	229995459	100.0%	121,1	126	0,979	106,2	5,7	99.1%
ERX2465188	1944815	242334002	100.0%	124,6	126	0,981	109,9	6,1	98.7%
ERX2465278	2083063	255592943	100.0%	122,7	126	0,95	112	6,4	98.5%
ERX2465662	1088402	133576015	100.0%	122,7	126	0,975	60	4,2	98.8%
ERX2465337	2000000	239906167	100.0%	120	126	0,953	101,6	9,4	98.6%
ERX2465453	2000000	241039378	100.0%	120,5	126	0,982	110	5,6	98.6%
ERX2465647	2000000	243244813	100.0%	121,6	126	0,978	106,7	7,1	98.5%
ERX2465186*	2280983	270693764	100.0%	118,7	126	0,352	44,8	3,2	98.7%
ERX2465307	2000000	243333574	100.0%	121,7	126	0,982	109,9	6,3	98.7%
ERX2465162	2000000	237028396	100.0%	118,5	126	0,982	106,5	7	98.5%
ERX2465577	2000000	235950053	100.0%	118	126	0,965	106,4	5	98.3%
ERX2465504	2000000	232831249	100.0%	116,4	126	0,775	84	5,2	98.6%

ERX2465623	2000000	238804378	100.0%	119,4	126	0,983	107,6	6,4	98.6%
ERX2465394	2000000	240479933	100.0%	120,2	126	0,98	109,2	6,4	98.7%
ERX2465360	2000000	238647229	100.0%	119,3	126	0,976	108,1	6	98.7%
ERX2465641	2000000	244700766	100.0%	122,4	126	0,976	110,3	6,4	98.6%
ERX2465597	2000000	240688723	100.0%	120,3	126	0,981	109,8	6,1	98.8%
ERX2465516	2000000	234841737	100.0%	117,4	126	0,87	95,2	5,7	98.4%
ERX2465438*	1999999	236401266	100.0%	118,2	126	0,457	50,8	3,5	98.6%
ERX2465689	2000000	232775777	100.0%	116,4	126	0,978	104,9	5,9	98.4%
ERX2465652	2000000	241386902	100.0%	120,7	126	0,975	106,8	7,5	98.6%
ERX2465466	1951823	236134097	100.0%	121	126	0,973	106,8	5,4	99.0%
ERX2465646	1560679	176440897	100.0%	113,1	126	0,977	79,3	5,3	98.3%
ERX2465215	2000000	233435914	100.0%	116,7	126	0,981	96,3	8,3	98.6%
ERX2465234	1999999	239164880	100.0%	119,6	126	0,979	108,6	6,5	98.7%
ERX2465534*	2000000	233015923	100.0%	116,5	126	0,06	6,2	1,8	98.9%
ERX2465614*	1999999	238807317	100.0%	119,4	126	0,015	1,6	1,4	99.0%
ERX2465583	2000000	241600924	100.0%	120,8	126	0,977	108,8	6,5	98.7%
ERX2465200*	1999997	227102980	100.0%	113,6	126	0,436	47,7	3,6	98.7%
ERX2465391	2000000	236196749	100.0%	118,1	126	0,977	104,8	7,3	98.6%
ERX2465449	1549731	181321530	100.0%	117	126	0,976	81,4	5,2	98.5%
ERX2465588	2000000	240096766	100.0%	120	126	0,978	107,4	6,6	98.8%
ERX2465242	1917623	227903087	100.0%	118,8	126	0,969	101,3	6,7	98.6%
ERX2465231	1757641	207149771	100.0%	117,9	126	0,979	92,4	6,1	98.6%
ERX2465568	1508146	178272438	100.0%	118,2	126	0,822	67,6	4,8	98.4%
ERX2465625	1650333	193771289	100.0%	117,4	126	0,98	82,8	6,7	98.5%
ERX2465315	1889054	222972166	100.0%	118	126	0,977	98,1	6,8	98.6%
ERX2465543	1769744	208261650	100.0%	117,7	126	0,916	87,7	6,1	98.7%
ERX2465615	1757188	206471466	100.0%	117,5	126	0,98	91,5	6,6	98.8%
ERX2465286	1995162	231332087	100.0%	115,9	126	0,978	102,4	6,4	98.2%
ERX2465485	1999999	231903909	100.0%	116	126	0,979	104,8	6,4	98.8%
ERX2465492	1999998	232634092	100.0%	116,3	126	0,966	103,5	7	98.5%
ERX2465221	2000000	224340008	100.0%	112,2	126	0,981	100,8	6,1	98.3%
ERX2465624	1997325	230095631	100.0%	115,2	126	0,971	103,7	4,9	98.6%
ERX2465343	2000000	238800285	100.0%	119,4	126	0,974	108,4	4,8	98.8%
ERX2465190	2000000	244240848	100.0%	122,1	126	0,947	105,2	7	98.8%
ERX2465169	2000000	249521349	100.0%	124,8	126	0,977	113,9	5,1	98.8%
ERX2465331	2000000	248347041	100.0%	124,2	126	0,978	113,5	5,1	98.7%
ERX2465268	2000000	237624196	100.0%	118,8	126	0,961	106	5,2	98.3%
ERX2465631	1424320	170828213	100.0%	119,9	126	0,968	76,4	4,5	98.8%
ERX2465522*	1999999	247116851	100.0%	123,6	126	0,099	11,4	1,8	98.7%
ERX2465357	2000000	247755094	100.0%	123,9	126	0,976	113,5	5,1	98.8%
ERX2465567	2000000	248208506	100.0%	124,1	126	0,978	113,8	5,6	98.3%
ERX2465276	2000000	239921512	100.0%	120	126	0,826	92,3	5,6	98.4%
ERX2465473	2000000	245230930	100.0%	122,6	126	0,979	110	6,6	98.4%
ERX2465539	2000000	247735636	100.0%	123,9	126	0,977	113,3	6,3	99.1%

*These isolates were in the Research Center Borstel (MTBseq) pipeline reported as likely contaminated with non-mycobacterial DNA, as indicated from the detection of low frequency variants.

Supplementary Table S3. Patient characteristics of 527/535 patients with complete data from the Netherlands Tuberculosis Register. This table was published in an earlier study [Jajou *et al*. Epidemiological links between tuberculosis cases identified twice as efficiently by whole genome sequencing than conventional molecular typing: A population-based study. Plos One. 2018;13(4):e0195413]. This supplementary material is hosted by *Eurosurveillance* as supporting information alongside the article [Towards standardisation: comparison of five whole genome sequencing (WGS) analysis pipelines for detection of epidemiologically linked tuberculosis cases], on behalf of the authors, who remain responsible for the accuracy and appropriateness of the content. The same standards for ethics, copyright, attributions and permissions as for the article apply. Supplements are not edited by *Eurosurveillance* and the journal is not responsible for the maintenance of any links or email addresses provided therein.

	**Study population (n = 527)**
**Age in years, median (range)**	35 (0 – 102)
**Age in categories (in years)**	
0-24	127 (23.7%)
25-44	215 (40.8%)
45-64	105 (19.9%)
65+	80 (15.2%)
**Gender, male**	316 (60%)
**Rural living**	373 (70.8%)
**Diagnosis**	
PTB	255 (48.4%)
ETB	200 (38%)
PTB+ETB	72 (13.7%)
**Resistance**	
Isoniazid mono-resistance	30 (5.7%)
Rifampicin mono-resistance	2 (0.4%)
Pyrazinamide mono-resistance	15 (2.8%)
Multidrug-resistant	12 (2.3%)
**Ethnicity**	
Dutch	65 (12.3%)
First generation migrant [a]	413 (78.4%)
Second generation migrant [b]	36 (6.8%)
Unknown	13 (2.5%)
**Risk group**	
Contact of tuberculosis patient	41 (7.8%)
Immigrant [c]	35 (6.6%)
Asylum seeker [d]	106 (20.1%)
Undocumented migrant	21 (4%)
Homeless	14 (2.7%)
Alcohol addict	4 (0.8%)
Drug addict	6 (1.1%)
Prisoner	11 (2.1%)
Travel to endemic regions > 3 months	15 (2.8%)
**Comorbidity**	
Diabetes	28 (5.3%)
Malignancy	19 (3.6%)
Renal failure	8 (1.5%)
Organ transplantation	2 (0.4%)
**Lineages [e]**	
EAI	60 (11.2%)
Beijing	43 (8.3%)
Delhi/CAS	117 (22.5%)
EAS	127 (24.4%)
LAM	57 (11%)
Cameroon	4 (0.8%)

Haarlem	78 (15%)
S-type	12 (2.3%)
TUR	2 (0.4%)
Uganda	3 (0.6%)
Ural	7 (1.3%)
West African 1	1 (0.2%)
West African 2	2 (0.4%)
No lineage assigned by PhyResSe	7 (1.3%)

PTB: pulmonary tuberculosis; ETB: extra-pulmonary tuberculosis; EAI: East-African-Indian; CAS: Central-Asian; EAS: Euro-American; LAM: Latin American-Mediterranean

[a] This is in the Netherlands Tuberculosis Register defined as a person was foreign-born and at least one parent was foreign-born.

[b] This is in the Netherlands Tuberculosis Register defined as a person born in the Netherlands, of whom at least one parent was foreign-born.

[c] This is in the Netherlands Tuberculosis Register defined as a person with a legal residence status other than a tourist or refugee/asylum seeker, who is subject to the immigrant screening regulations and who resides in the Netherlands < 2.5 years.

[d] This is in the Netherlands Tuberculosis Register defined as a person who is subject to regulations relating to the screening of asylum seekers, already has a valid residence status as an asylum seeker or is still in the asylum seeker procedure and has been residing in the Netherlands < 2.5 years.

[e] These data are (RIVM) laboratory data and were available for all 535 isolates of which 520 isolates belong to *M. tuberculosis*.

Supplementary Table S4. Sequences excluded by one or more pipelines. Xs indicate a sequence was excluded in the respective pipeline. This supplementary material is hosted by *Eurosurveillance* as supporting information alongside the article [Towards standardisation: comparison of five whole genome sequencing (WGS) analysis pipelines for detection of epidemiologically linked tuberculosis cases], on behalf of the authors, who remain responsible for the accuracy and appropriateness of the content. The same standards for ethics, copyright, attributions and permissions as for the article apply. Supplements are not edited by *Eurosurveillance* and the journal is not responsible for the maintenance of any links or email addresses provided therein.

	RIVM SNP	Oxford University SNP	Research Center Borstel (MTBseq) SNP	cgMLST (allele)	SSI SNP
ERX2465667	X	X	X	X	X
ERX2465614	X		X	X	X
ERX2465534	X	X	X		X
ERX2465514	X	X	X	X	X
ERX2465557	X		X	X	X
ERX2465171	X		X	X	X
ERX2465192	X		X		X
ERX2465522	X		X		X
ERX2465498	X		X		X
ERX2465621	X		X		X
ERX2465451			X		
ERX2465664				X	
ERX2465483				X	X
ERX2465610		X			
ERX2465299					X
ERX2465354					X
ERX2465455		X			
ERX2465589		X			
Total excluded	10[a]	6[b]	11[c]	7[d]	13[e]

[a] Sequences were excluded due to a mean coverage depth < 20x.

[b] Sequences did not pass the pipeline for several reasons based on expert's opinion.

[c] Sequences excluded due to insufficient coverage depth < 30x or breadth < 80%, and/or detection of considerable contamination by non-MTBC sequence.

[d] Sequences excluded due to < 90% of the genes in the cgMLST scheme passing quality criteria.
[e] Sequences excluded due to at least 95% of the H37Rv reference genome (not including repetitive regions) was required to be above 10x coverage depth.
