# Supplementary material for: Towards standardisation: comparison of five whole genome sequencing (WGS) analysis pipelines for detection of epidemiologically linked tuberculosis cases
Source: Euro Surveill. 2019 Dec 12;24(50):1900130. doi: 10.2807/1560-7917.ES.2019.24.50.1900130 (PMC6918587; doi:10.2807/1560-7917.ES.2019.24.50.1900130)
Supplement: Supplementary Material S1 [file 19-00130_SupplementaryMaterialS1.zip › SupplementaryMaterialS1_Disclaimer.pdf]

Supplementary Material S1 Disclaimer

This supplementary material is hosted by *Eurosurveillance* as supporting information alongside the article [Towards standardisation: comparison of five whole genome sequencing (WGS) analysis pipelines for detection of epidemiologically linked tuberculosis cases], on behalf of the authors, who remain responsible for the accuracy and appropriateness of the content. The same standards for ethics, copyright, attributions and permissions as for the article apply. Supplements are not edited by *Eurosurveillance* and the journal is not responsible for the maintenance of any links or email addresses provided therein.
